# Radio‐Activated Selenium‐Doped Janus Ag/Ag_2_Se_x_S_y_ Nanoparticles for Precise Cancer NIR‐II Fluorescence Imaging and Radiosensitization Therapy

**DOI:** 10.1002/advs.202417828

**Published:** 2025-04-17

**Authors:** Kang Zhu, Zhanyuan Li, Jingjing Cao, Yixi Cao, Jimei Wang, Shiyu Wang, Ling Chen, Huiqin Zhou, Wei Huang, Hanxun Zou, Qunsheng Li, Jing Mu, Jibin Song

**Affiliations:** ^1^ State Key Laboratory of Chemical Resource Engineering College of Chemistry College of Chemical Engineering Beijing University of Chemical Technology Beijing 100029 P. R. China; ^2^ Shandong Cancer Hospital and Institute Shandong First Medical University and Shandong Academy of Medical Sciences Department of Radiation Oncology Jinan 250117 P. R. China; ^3^ School of Materials Science and Engineering University of Jinan Jinan 250022 P. R. China; ^4^ Fujian Provincial Key Laboratory of Ecology‐Toxicological Effects & Control for Emerging Contaminants Key Laboratory of Ecological Environment and Information Atlas College of Environmental and Biological Engineering Putian University Putian 351100 P. R. China; ^5^ Institute of Precision Medicine Peking University Shenzhen Hospital Shenzhen 518036 P. R. China

**Keywords:** bioimaging, janus nanoparticle, NIR‐II fluorescence imaging, radiotherapy, X‐ray

## Abstract

The efficacy of radiotherapy (RT) is often limited by insufficient tumor selectivity and suboptimal therapeutic responses. To overcome these problems, a new kind of selenium‐doped Ag/Ag_2_S Janus nanoparticles (Ag/Ag_2_Se_x_S_y_ JNPs) is presented as radio‐responsive molecular probes for precise tumor imaging and enhanced radiosensitization. By adjusting the selenium precursor input, heterojunction nanoparticles with tunable doping ratios are synthesized, optimizing X‐ray absorption and energy storage properties. Upon X‐ray irradiation, the Ag/Ag_2_Se_x_S_y_ JNPs interact with overexpressed hydrogen peroxide (H_2_O_2_) in tumor cells, generating highly toxic hydroxyl radicals (·OH), which effectively induce tumor cell apoptosis. Additionally, Selenium incorporation improves electron–hole pair separation efficiency and enhances the photocurrent response, promoting increased electron transfer and ·OH generation, thus amplifying reactive oxygen species (ROS) production and enhancing radiosensitization. Furthermore, the fluorescence “OFF‐ON” mechanism, triggered by H_2_O_2_‐induced etching of silver allows real‐time monitoring of H_2_O_2_ levels via the second near‐infrared window (NIR‐II) fluorescence (FL) imaging “Turn On”, which delineates tumor boundaries for precise RT and reduce side effects to normal tissue. This dual‐functional platform not only enables real‐time tracking but also enhances therapeutic outcomes, offering a promising approach to precision cancer treatment.

## Graphical abstract

A new kind of selenium‐doped Ag/Ag_2_S Janus nanoparticles (Ag/Ag_2_Se_x_S_y_ JNPs) is reported as radio‐responsive probes for specifically hydrogen peroxide (H_2_O_2_)‐activated cancer imaging and enhanced radiosensitization, offering a promising approach for precision cancer theranostics.

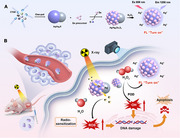

## 1 Introduction

Radiotherapy (RT), as an important therapeutic tool in clinic, is widely employed in the treatment of many diseases, especially malignant tumors.^[^ ^1^ ^]^ Its basic principle is to precisely target tumor cells through high‐energy X‐rays to destroy the DNA structure, thus inhibiting cell proliferation and differentiation.^[^ ^2^ ^]^ However, the unwanted radiotherapy tolerance and resistance increases the risk of tumor recurrence and metastasis, which leads to unsatisfactory radiotherapy results and poor prognosis.^[^ ^3^ ^]^ Therefore, efficient and safe radiosensitizers for increasing reactive oxygen species (ROS) generation need to be developed to enhance the effectiveness and efficiency of radiotherapy in cancer treatment.^[^ ^4^ ^]^ Significantly, most of the current radiosensitizers are not biologically responsive, such as gold or hafnium oxide materials.^[^ ^5^ ^]^ The development of ideal alternatives with the capability to be specifically activated in tumor site is highly demanded for optimum enhancement of RT.^[^ ^6^ ^]^

On the other hand, precision radiotherapy relies on high‐precision imaging guidance to ensure accurate tumor localization and boundary discrimination during treatment.^[^ ^7^ ^]^ At present, the clinical precision radiotherapy mainly relies on Computed Tomography (CT) to delineate the tumor radiotherapy area, which often suffers from poor resolution and lack of precise functional information.^[^ ^8^ ^]^ Radiotherapy molecules based on tumor marker responsiveness can improve the accuracy of tumor diagnosis and delineation of tumor boundaries, and help clinicians to plan the radiation dose and irradiation exposure range more accurately before radiotherapy, thereby greatly reducing damage to normal tissues.^[^ ^9^ ^]^ Optical imaging, especially in the second near‐infrared window (NIR‐II, 1000–1700 nm), has the advantages of high spatial resolution and deep penetration, which can provide more accurate and comprehensive biological information by monitoring the physiological and pathological processes in organisms, and has great potential in disease diagnosis.^[^ ^10^ ^]^ Therefore, it is urgent to develop radiation‐responsive radiosensitizers and bio‐responsive tumor fluorescence imaging molecular probes, which are of great scientific significance and clinical utility for achieving precision radiotherapy.^[^ ^11^ ^]^

In recent years, Janus Ag/Ag_2_S nanoparticles have emerged as promising candidates for diverse biological applications. their unique asymmetrical structure, coupled with excellent optical and electrochemical properties make them as highly effective agents in bioimaging,^[^ ^12^ ^]^ cancer treatment,^[^ ^13^ ^]^ and antimicrobial applications,^[^ ^14^ ^]^ etc. For example, Janus Ag/Ag_2_S nanoparticles enable high‐resolution imaging of deep tissues due to their superior NIR fluorescence imaging capabilities. In addition, their efficient photothermal conversion performance and photodynamic effect have been harnessed in photothermal therapy (PTT) and photodynamic therapy (PDT). Furthermore, these nanoparticles have been used as drug carriers to achieve targeted drug delivery, significantly improving the efficacy of cancer treatment.

**Figure 1:**
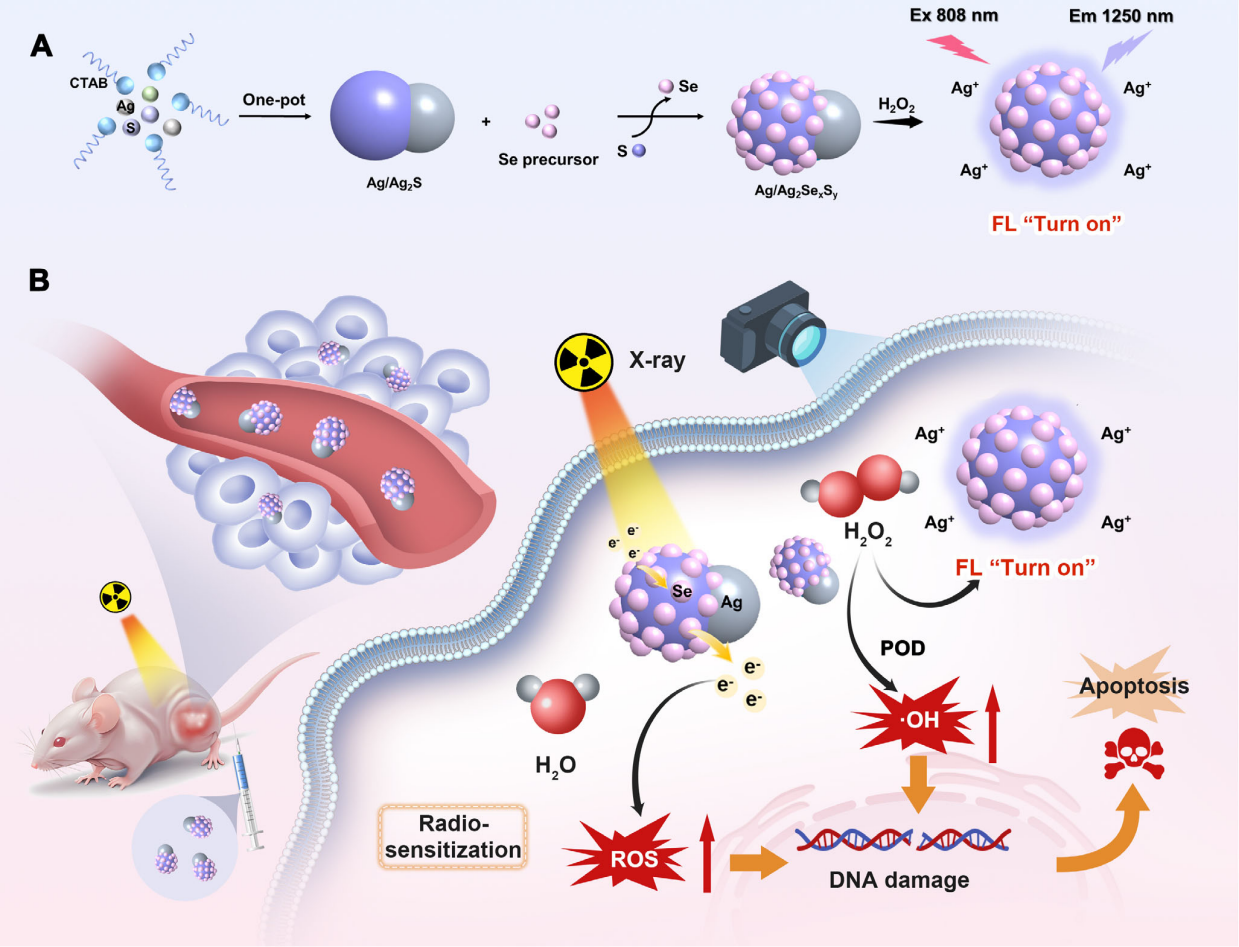
Schematic illustration of (A) preparation of Ag/Ag_2_Se_x_S_y_ JNPs, and (B) its application for tumor biomarker H_2_O_2_ responsive NIR‐II fluorescence imaging‐guided radiosensitization of precision cancer radiotherapy.

Herein, we developed a selenium (Se)‐doped radio‐responsive molecular probe, Ag/Ag_2_Se_x_S_y_ Janus nanoparticles (JNPs), for monitoring tumor hydrogen peroxide (H_2_O_2_) levels and sensitizing therapeutic effects during RT (**Figure** **1**). Heterojunction nanoparticles with different doping ratios were synthesized by modulating the amount of Se precursor. Se, with higher atomic number than sulfur, can significantly enhance the absorption of X‐rays and store energy. The nanoparticles can generate electron–hole pairs under X‐ray irradiation, whose electrons can be delivered to the overexpressed H_2_O_2_ in the tumor microenvironment to generate highly toxic hydroxyl radicals (·OH), which in turn kill the tumor cells. The doping of Se improves the separation efficiency of electron–hole pairs with a stronger photocurrent response, resulting in more electron transfer.^[^ ^15^ ^]^ In addition, the heterogeneous structure improves charge separation and facilitates the generation of more ·OH in tumor cells.^[^ ^16^ ^]^ Ultimately, the Ag/Ag_2_Se_x_S_y_ nano‐heterojunction acts as an efficient radiosensitizer that promotes the overproduction of ROS in tumor cells, which in turn induces intracellular DNA damage in tumor cells and effectively enhances radiation‐induced apoptosis. In addition, precision radiotherapy requires imaging guidance. The level of H_2_O_2_ is monitored by NIR‐II FL imaging. Under 808 nm laser irradiation, electrons in the Ag fraction were transferred to the Ag_2_S fraction, which compensated for the electron defects in the Ag_2_S fraction and quench its fluorescence. The Ag was etched by tumor overexpression of H_2_O_2_, which blocked the electron transfer process and restored the electron‐deficient state of Ag_2_S, thus activating NIR‐II fluorescence. The H_2_O_2_‐activated NIR‐II fluorescence imaging platform was employed to track radiotherapy molecules in real time and delineate tumor radiotherapy regions. The molecular probes integrating radiation‐responsive radiosensitization and bio‐responsive tumor fluorescence imaging hold great potential for achieving precision radiotherapy.

## 2 Results and Discussion

### 2.1 Preparation and Characterization of Ag/Ag_2_Se_x_S_y_ JNPs

**Figure 2:**
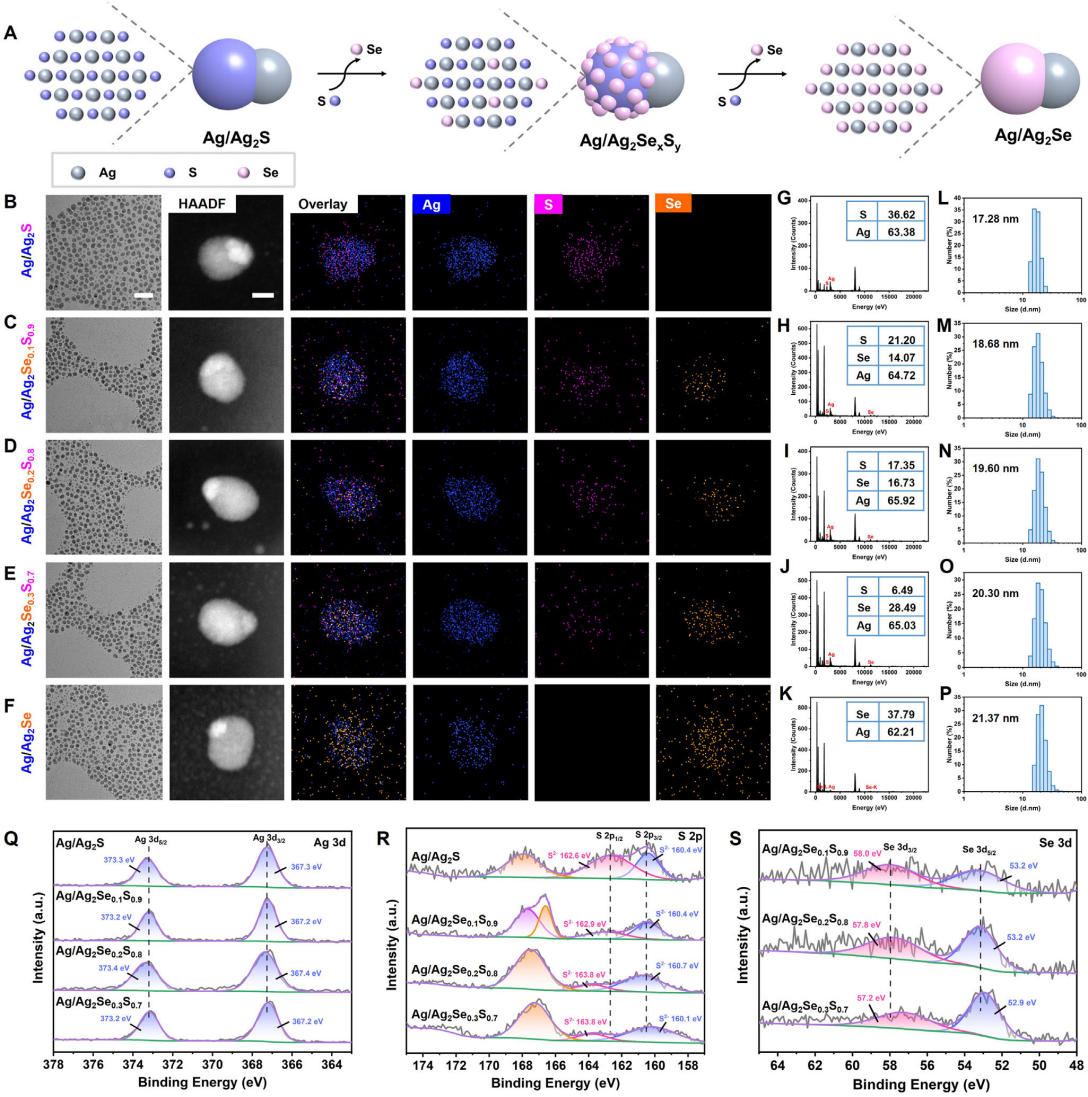
A) Schematic illustration of the proposed doping route for preparation of Se‐doped Ag/Ag_2_Se_x_S_y_ JNPs. TEM images (scale bar: 50 nm), high‐angle annular dark‐field (HAADF) imaging and element mapping (scale bar: 5 nm) (B–F), corresponding elements surface scanning (EDS) analysis (G–K), hydrodynamic diameter distributions (L–P) and XPS analysis (Q–S) of JNPs with different Se‐doping ratios.

To prepare Ag/Ag_2_Se_x_S_y_ JNPs, the Se precursor (Na_2_SeSO_3_) was first obtained by reacting Se powder with Na_2_SO_3_. Subsequently, asymmetric Ag/Ag_2_Se_x_S_y_ JNPs were successfully prepared by a modified one‐pot method using silver nitrate (AgNO_3_) as the silver source, thioacetamide (TAA) and Na_2_SeSO_3_ as the sulfur and selenium sources, respectively (where x, y represents the percentage of selenium and sulfur, respectively). By adjusting the molar ratios of selenium and sulfur sources without changing additional conditions, the precise control of the selenium content in Ag/Ag_2_Se_x_S_y_ JNPs was achieved, which were labeled as Ag/Ag_2_Se_0.1_S_0.9_, Ag/Ag_2_Se_0.2_S_0.8_, and Ag/Ag_2_Se_0.3_S_0.7_ JNPs, respectively (**Figure** **2**A). Based on the reported facile preparation strategy, we can synthesize Ag/Ag_2_Se_x_S_y_ JNPs with various of Se content to face the diverse of applications. Transmission electron microscopy (TEM) results showed that the JNPs with different Se doping ratios exhibit snowman‐like shape with uniform particle size and good dispersion (Figure 2B–F). High‐angle annular dark field (HAADF) scanning transmission electron microscopy (STEM) and elemental mapping verified the heterojunction nanostructure of Ag/Ag_2_Se_x_S_y_ and the successful doping of Se elements.

In addition, the elemental distribution of Se in the Ag_2_Se_x_S_y_ fraction gradually increases proportionally with the feeding ratio of Se and S, indicating that the Se content can be controlled by adjusting the feeding ratio of Se and S. The elements surface scanning (EDS) analysis shows that the atomic percentage of Ag remains nearly constant, while the atomic percentages of Se are ≈14.07%, 16.73%, and 28.49% in the three JNPs, respectively (Figure 2G–K). The average hydrodynamic diameter of Ag/Ag_2_Se_0.1_S_0.9_, Ag/Ag_2_Se_0.2_S_0.8_, and Ag/Ag_2_Se_0.3_S_0.7_ JNPs were 18.68, 19.60, and 20.30 nm, respectively, in the dynamic light scattering (DLS) results (Figure 2L–P). The peak shapes in the UV–vis absorption spectra of different JNPs at the same concentration were almost consistent. It was observed that the absorbance of JNPs at 808 nm showed a slight decrease with the increase ratio of the doped selenium (Figure S1, Supporting Information). The change of nanoparticle sizes with Se doping may be attributed to the substitution of smaller S atoms by larger Se atoms, leading to a gradual increase in the particle size. X‐ray diffraction (XRD) analysis of Ag/Ag_2_Se_x_S_y_ JNPs revealed obvious diffraction peaks of Ag_2_Se, especially in samples with higher Se‐doping ratios (Figure S2, Supporting Information). In addition, we determined the elemental signals of the JNPs by X‐ray photoelectron spectroscopy (XPS) (Figure 2Q–S). The Ag 3d spectrum demonstrated the coexistence of Ag^0^ and Ag^+^ in the JNPs. Compared with that of Ag/Ag_2_S, the decrease of elemental S in the S 2p spectrum and the increase of elemental Se in the Se 3d spectrum with the doping of Se further confirmed the successful doping of Se. In addition, the change of surface charge of JNPs from positive to negative electricity proved the successful modification of PEG (Figure S3, Supporting Information). No obvious aggregation was observed after 30 days storage, indicating their excellent water solubility and stability (Figure S4, Supporting Information).

### 2.2 H_2_O_2_‐Activated NIR‐II FL Imaging and Radiosensitization of Ag/Ag_2_Se_x_S_y_ JNPs

**Figure 3:**
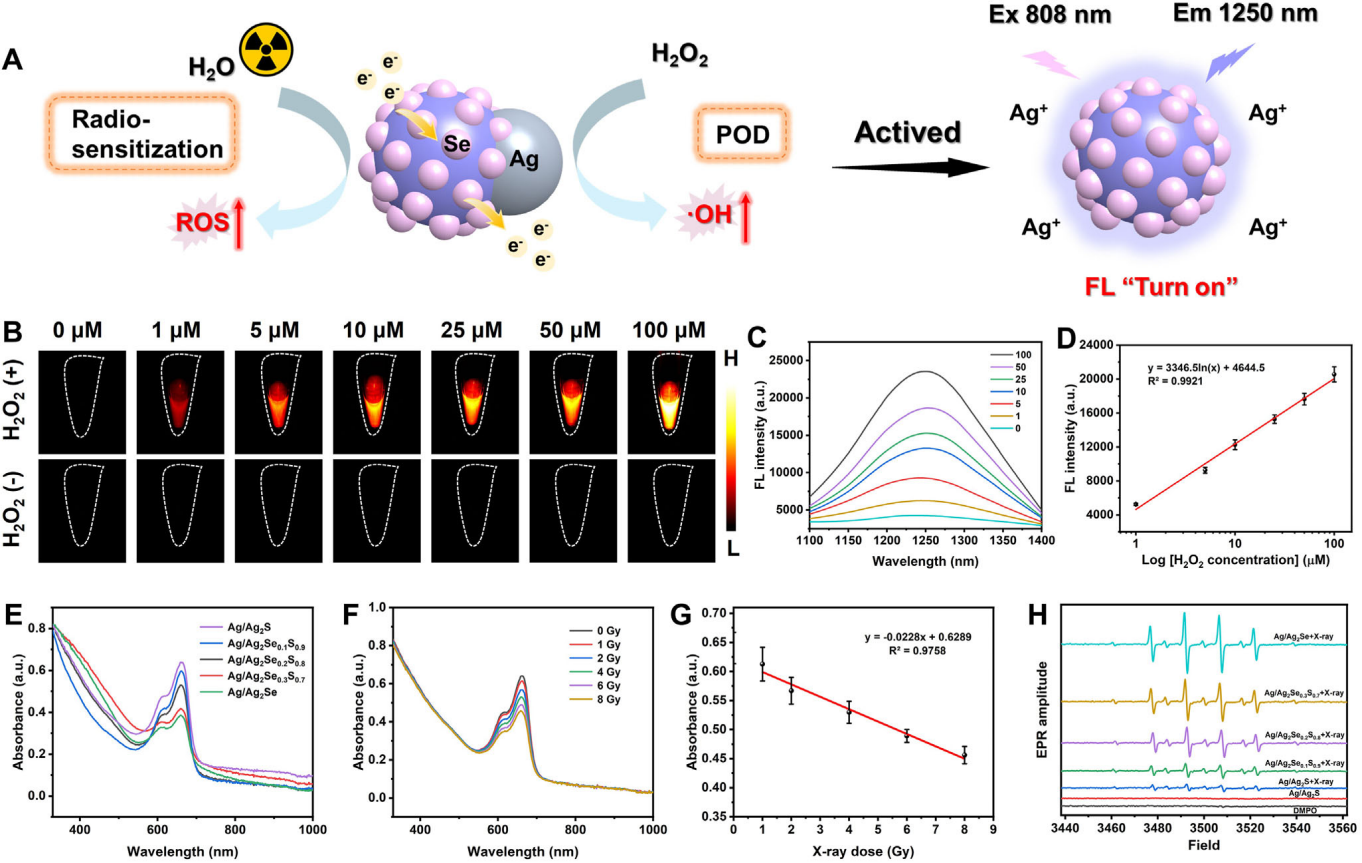
A) Schematic diagram of H_2_O_2_‐activated NIR‐II FL imaging of JNPs and their radiosensitization to promote ROS production. B) NIR‐II FL images of JNPs at 1250 nm after treatment with/without different concentrations of H_2_O_2_. C) NIR‐II FL spectra of JNPs treated with different concentrations of H_2_O_2_ under 808 nm laser irradiation. D) Correlation of FL intensity at 1250 nm in the FL spectra and H_2_O_2_ concentration (*n* = 6). E) The degradation of MB after reacted with JNPs under X‐ray irradiation. F) The degradation of MB after reacted with Ag/Ag_2_Se_0.2_S_0.8_ exposed with different doses of X‐ray. G) Correlation between X‐ray dose and absorbance of MB at wavelength of 660 nm. H) ESR spectroscopy of •OH of different kinds of JNPs after X‐ray irradiation.

Ag_2_S quantum dots (QDs) exhibit excellent NIR‐II FL imaging properties, mainly attributing to their electronic defects, which plays an important role in affecting the luminescence behavior of QDs.^[^ ^17^ ^]^ Thus, by introducing plasmonic Ag NP to one surface of Ag_2_S QD, the designed Janus Ag/Ag_2_S QDs show non‐fluorescence behavior due to the presence of plasmonic Ag part. The mechanism involves electron migration from plasmonic Ag to Ag_2_S nanoparticles (NPs) under laser irradiation, which fills the electronic defects in Ag_2_S and triggers a burst in its FL emission. When Ag was converted to Ag^+^ by H_2_O_2_ etching, the electron transfer between the plasmonic Ag and Ag_2_S NPs was interrupted, restoring the electron‐deficient state of Ag_2_S and thus activating NIR‐II FL (**Figure** **3**A). The disappearance of the Ag portion in the elemental mapping (Figure S5, Supporting Information) and the reduction of the particle size (≈12 nm) in the DLS (Figure S6, Supporting Information) confirmed the Ag etching. Interestingly, different selenium‐doped JNPs exhibited diverse degrees of FL recovery under the same conditions. The degree of NIR‐II FL recovery is weaker with increasing selenium doping (Figure S7, Supporting Information). We therefore chose Ag/Ag_2_Se_0.2_S_0.8_ JNPs as representative particles for subsequent experiments. NIR‐II FL images revealed that JNPs interacting with H_2_O_2_ showed a bright FL imaging at 1250 under 808 nm laser irradiation (Figure 3B) and the FL spectra showed strong FL emission in the range of 1100 – 1400 nm (Figure 3C). While in the control group without H_2_O_2_, JNPs maintained the FL off state.

Investigating the impact of varying concentrations (0–100 µm) of H_2_O_2_ on the FL enhancement of JNPs revealed a significant linear relationship between the NIR‐II FL intensity of JNPs and the concentration of H_2_O_2_, with a low detection limit (0.3 µm) (Figure 3D). In addition, compared to other reactive oxygen species (ROS), only H_2_O_2_ activated the NIR‐II FL of JNPs, indicating a high degree of specificity and selectivity (Figure S8, Supporting Information). These findings suggest that JNPs hold significant promise for tumor precision diagnosis by monitoring H_2_O_2_ levels.

The ability of Ag/Ag_2_Se_x_S_y_ JNPs to produce ·OH under X‐ray irradiation was examined by methylene blue (MB) experiments and electron spin resonance (ESR) spectroscopy.^[^ ^18^ ^]^ The JNPs with different Se‐doping ratios were mixed with MB, which was observed to gradually change from blue to colorless upon X‐ray irradiation, and its absorbance at a wavelength of 660 nm accordingly decreased (Figure 3E). This decreasing trend in absorbance became increasingly significant with the increase of Se‐doping or ray dose (Figure 3F) and showed a linear correlation (Figure 3G). Similarly, when mixed with 4,4,5,5‐dimethyl‐1‐pyrrolineN‐oxide (DMPO) solution (a ·OH scavenger), characteristic signal peaks of ·OH were detected by ESR spectroscopy after X‐ray irradiation (Figure 3H). This signal intensified with increasing Se‐doping, further validating the enhanced ability of Se‐doped JNPs to produce ·OH under X‐ray irradiation. Se‐doped JNPs combined with X‐rays effectively induced a robust generation of ROS, showing a synergistic enhancement benefit. Furthermore, we tested the MB degradation and ESR spectroscopy of ·OH by JNPs in the presence and absence of H_2_O_2_ and X‐ray irradiation, respectively (Figure S9, Supporting Information). The results showed that the POD‐like activity of JNPs produced a small part of ·OH, and most of the ·OH was generated by the radiosensitization of JNPs.

### 2.3 Study of the Radiosensitization Mechanism of Ag/Ag_2_Se_x_S_y_ JNPs under X‐Ray Irradiation

**Figure 4:**
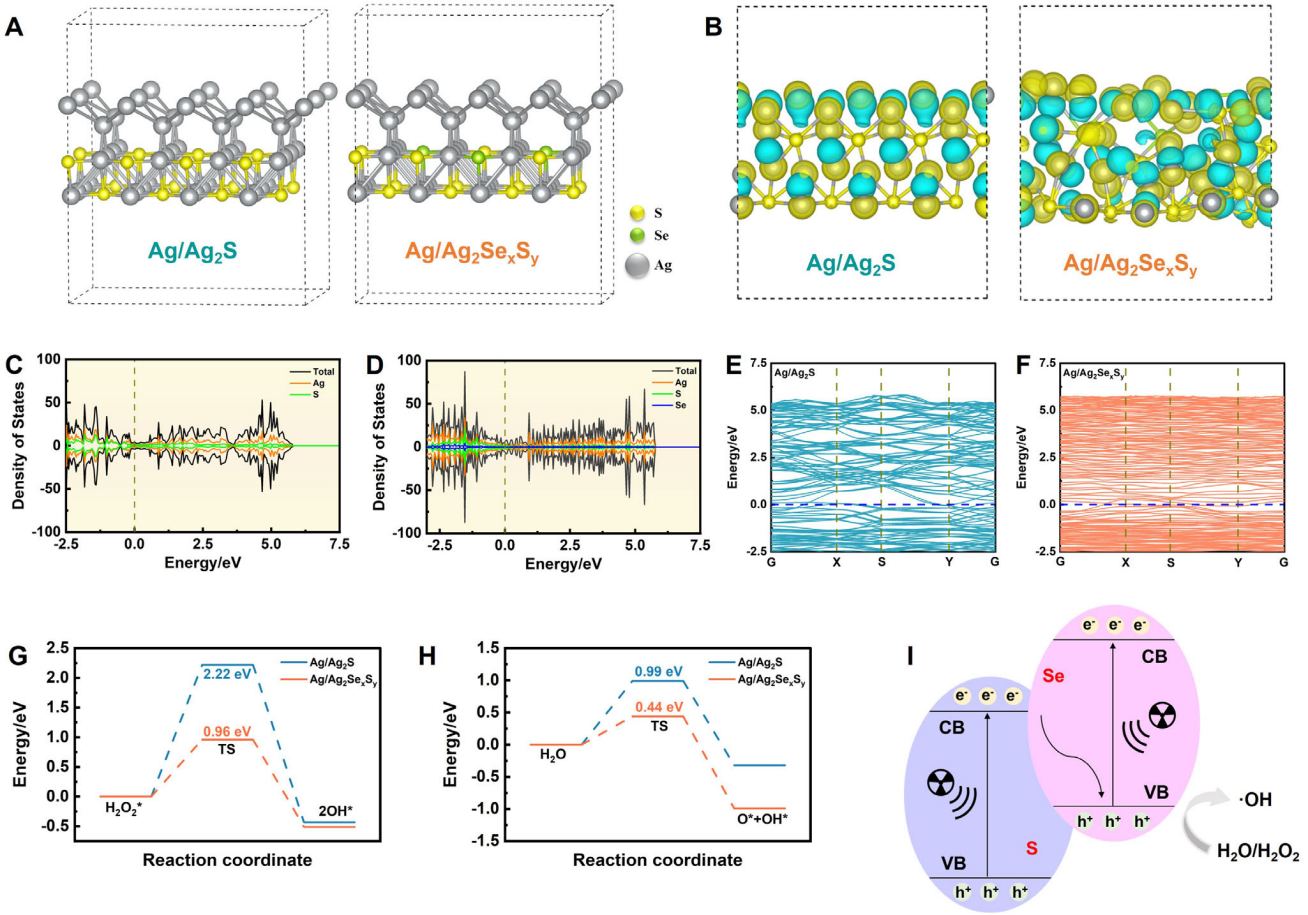
A) Janus Ag/Ag_2_S and Ag/Ag_2_Se_x_S_y_ DFT modeling. B) Differential charge densities of Ag/Ag_2_S and Ag/Ag_2_Se_x_S_y_. Densities of states of C) Ag/Ag_2_S and D) Ag/Ag_2_Se_x_S_y_. Energy bands of E) Ag/Ag_2_S and F) Ag/Ag_2_Se_x_S_y_. Reaction step diagram of dissociation G) H_2_O_2_ and H) H_2_O to ·OH of Ag/Ag_2_S and Ag/Ag_2_Se_x_S_y_. I) Schematic illustration of JNPs promoting electron–hole pair separation under X‐ray irradiation.

Se with high X‐ray absorption and energy storage properties was widely used as X‐ray activated materials.^[^ ^19^ ^]^ To explore the potential mechanisms of the radiosensitization by Ag/Ag_2_Se_x_S_y_ JNPs, DFT calculations were performed. Structural models were established based on the atomic configurations of Ag/Ag_2_S and Ag/Ag_2_Se_x_S_y_ (**Figure** **4**A). According to Figure 4B, Se doping decreases the interfacial charge transfer, while simultaneously increases the internal electron transfer. This reflects that Se doping promotes the electron transfer effect in the material and improves the electronic conductivity. Figure 4C,D reveals that Se doping leads to an increase in the total density of states. At the same time, electrons become denser near the Fermi level (0 eV), which suggests that more electrons in the material can participate in conduction. Figure 4E,F demonstrates a gradual increase in electron density between the top of the valence band and the bottom of the conduction band. The reduced energy required for electrons to cross from the valence band to the conduction band results in a higher likelihood of electronic transition. Remarkably, compared with Ag/Ag_2_S (2.22 eV), Ag/Ag_2_Se_x_S_y_ (0.96 eV) had a lower energy barrier, facilitating stronger H_2_O_2_ adsorption and superior peroxidase (POD)‐like catalytic activity (Figure 4G). DFT analyses also showed that the Se‐doped Ag/Ag_2_Se_x_S_y_ (0.44 eV) has lower reaction energy than Ag/Ag_2_S (0.99 eV) in the process of H_2_O dissociation on the surface, making it easier for H_2_O to produce ·OH (Figure 4H). These results emphasize that Se‐doped Ag/Ag_2_Se_x_S_y_ JNPs significantly enhance X‐ray absorption and energy storage by accelerating electron transfer and lowering energy barriers, thereby improving the separation efficiency of electron–hole pairs and facilitating increased ·OH production (Figure 4I). Overall, the Se‐doped Ag/Ag_2_Se_x_S_y_ JNPs has great potential for application in POD‐like catalysis and radiosensitization to produce ·OH.^[^ ^20^ ^]^ The direct relationship between the X‐ray energy absorption and storage promotes the separation efficiency of electron–hole pairs after X‐ray irradiation, which then enhances the sensitization effect of radiotherapy. Moreover, the Selenium doping in the JNPs also further improves the electron–hole pair separation efficiency on the surface of JNPs. Therefore, through regulating the heterostructure, the Se plays an important role in accelerating the electron cross to generate more ·OH.

### 2.4 In Vitro Evaluation of Radiosensitization and Activated NIR‐II FL Imaging

**Figure 5:**
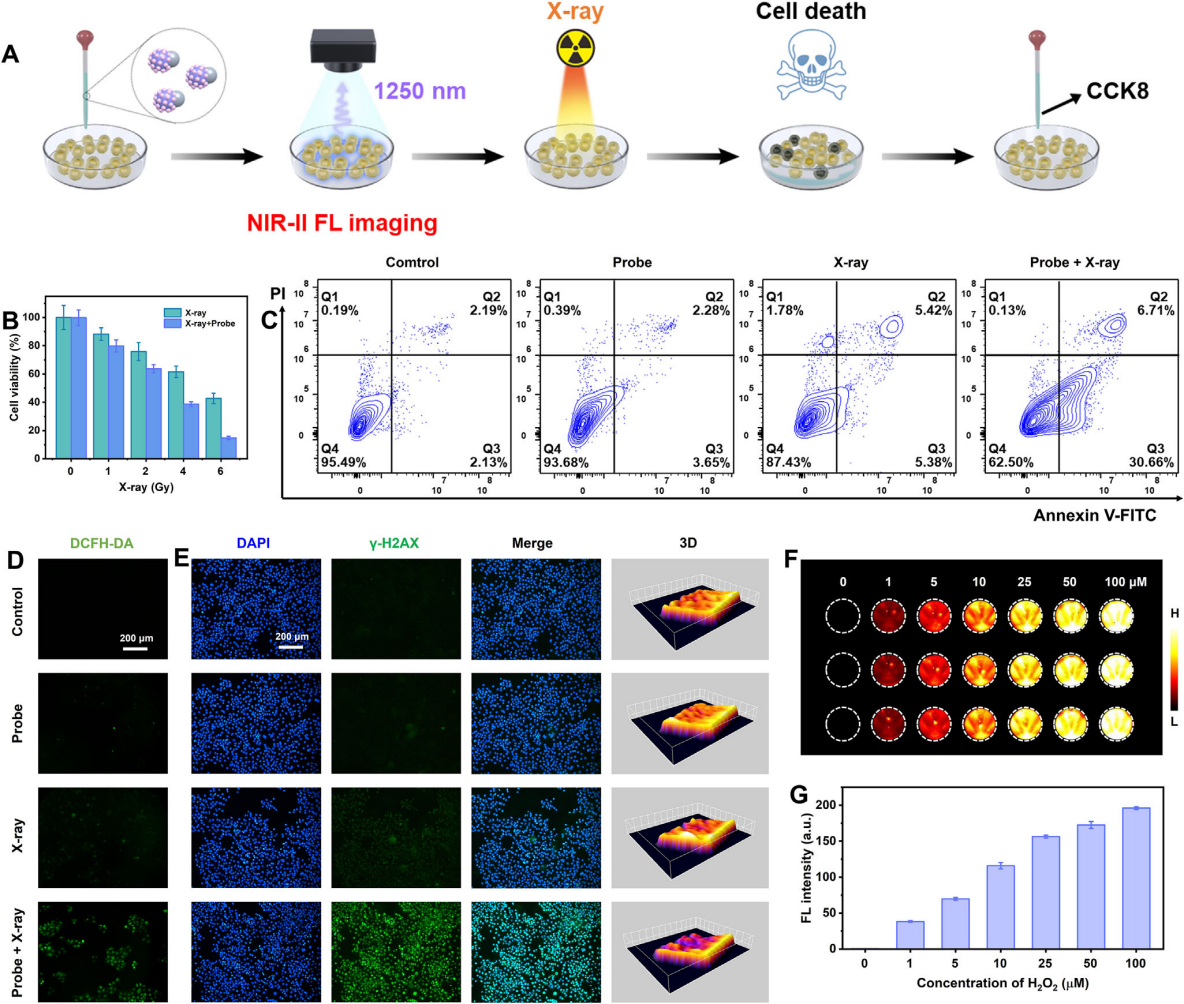
A) Schematic diagram of NIR‐II FL imaging for real‐time quantitative monitoring of intracellular H_2_O_2_ and in vitro evaluation of radiosensitization. B) Cell viability of cancer cells incubated with/without JNPs and then irradiated with different X‐ray doses. C) The flow cytometry analyses cell apoptosis of cancer cells induced by different treatments. D) Inverted fluorescence microscopy images of cancer cells stained with DCFH‐DA after different treatments to detect ROS generation. E) Inverted fluorescence microscopy images of cancer cells stained with DAPI, γ‐H2AX, and their merge and 3D imaging after different treatments to detect the extent of DNA damage. F) NIR‐II FL images of cancer cells treated with JNPs and different concentrations of H_2_O_2_ (*n* = 3). G) The corresponding NIR‐II FL intensity of (F).

In vitro experiments were performed to assess the radiosensitization of JNPs and their ability to monitor intracellular H_2_O_2_ levels (**Figure** **5**A). The biocompatibility of JNPs on human esophageal cancer cells kyse150 was first evaluated using Cell Counting Kit‐8 (CCK8 method). Even after 24 h of treatment with JNPs at a high concentration of 200 µg mL^−1^, the cell survival rate remained above 95%, indicating that JNPs have good biosafety (Figure S10, Supporting Information). Subsequently, we investigated the radiosensitization ability of JNPs on kyse150 tumor cells. The cell viability was detected under X‐ray irradiation at 0, 1, 2, 4, and 6 Gy radiation doses, respectively. The results showed a gradual decrease in cell viability with increasing radiation dose (Figure 5B). In the presence of probe (200 µg mL^−1^), the cell viability after X‐ray irradiation (6 Gy) at 24 h was further reduced (below 20%), leading to a significant increase in cell death. These results suggest that JNPs exhibit notable radiosensitization activity, which significantly enhances the therapeutic effect of RT.

In order to verify the radiosensitization and cancer cell killing abilities of JNPs on tumor cells, the cells were treated with different conditions, which were divided as Control group, Probe group, X‐ray group, and Probe + X‐ray group. Flow cytometry was performed to analyze the apoptosis of cells in different treatment groups (Figure 5C). After the cells treated with JNPs for 6h and irradiated with X‐ray (2 Gy), they were further incubated with cell culture medium for another 12 h. The cell viability results revealed no significant apoptosis in the cells of the Control and Probe groups. However, the apoptosis was significantly increased in the Probe + X‐ray group (37.37%) compared to the X‐ray group (10.8%). Typically, apoptosis triggered by X‐ray irradiation is closely associated with high levels of intracellular ROS generation. There was a correlation between the increase in apoptosis and the rise in intracellular ROS levels. Therefore, we utilized the fluorescent probe 2′,7′‐Dichlorodihydrofluorescein diacetate (DCFH‐DA) for ROS detection.^[^ ^21^ ^]^ The results showed a significant green fluorescence in the Probe + X‐ray group compared to the Control, Probe, and X‐ray group, indicating a substantial increase in intracellular ROS levels (Figure 5D). In addition, calcein AM and propidium iodide (PI) double staining experiments revealed a significant increase in the intensity of red fluorescence in the Probe + X‐ray group compared to the other groups, indicating that cell death was significantly induced by JNPs combined with radiotherapy (Figure S11, Supporting Information).

Based on the distinguished properties of JNPs in triggering ROS overproduction, we further briefly studied their radiosensitization mechanism in tumor cells. Intracellular DNA damage was detected by using γ‐H2AX immunofluorescence, in which γ‐H2AX (i.e., phosphorylated H2AX) is a common DNA damage marker (Figure 5E).^[^ ^22^ ^]^ Compared with the Control, Probe, and X‐ray groups, the Probe + X‐ray group showed significantly enhanced green fluorescence in the nucleus, indicating more DNA damaged. This phenomenon verified that JNPs enhanced the therapeutic effects of RT by damaging DNA in the nucleus. On the other hand, the biocompatibility of JNPs was evaluated by hemolysis experiments, and we found that JNPs had good blood compatibility (Figure S12, Supporting Information). Even at a concentration of 200 µg mL^−1^, the rate of JNPs‐induced hemolysis was low (no more than 10%), comparable to the negative control. Taken together, these results suggest that Se‐doped nano‐heterojunctions promote ROS overproduction and DNA damage under X‐ray irradiation, which in turn induces apoptosis of tumor cells to enhance the biochemical sensitivity of radiotherapy, and ultimately achieves radiosensitization.

NIR‐II FL images were collected on 96‐well plates after incubating cells with JNPs and different concentrations of H_2_O_2_. With the increase of H_2_O_2_ concentration, the NIR‐II FL signals of the cells were gradually enhanced at 1250 under 808 nm laser irradiation (Figure 5F,G). It was interesting that the cancer cells biomarker, H_2_O_2,_ could etch the Ag part and recover the NIR‐II FL imaging of JNPs. Thus, we can then further apply the JNPs to image the H_2_O_2_ in vivo and diagnosis of tumor.

### 2.5 In Vivo H_2_O_2_‐Activated NIR‐II FL Imaging

**Figure 6:**
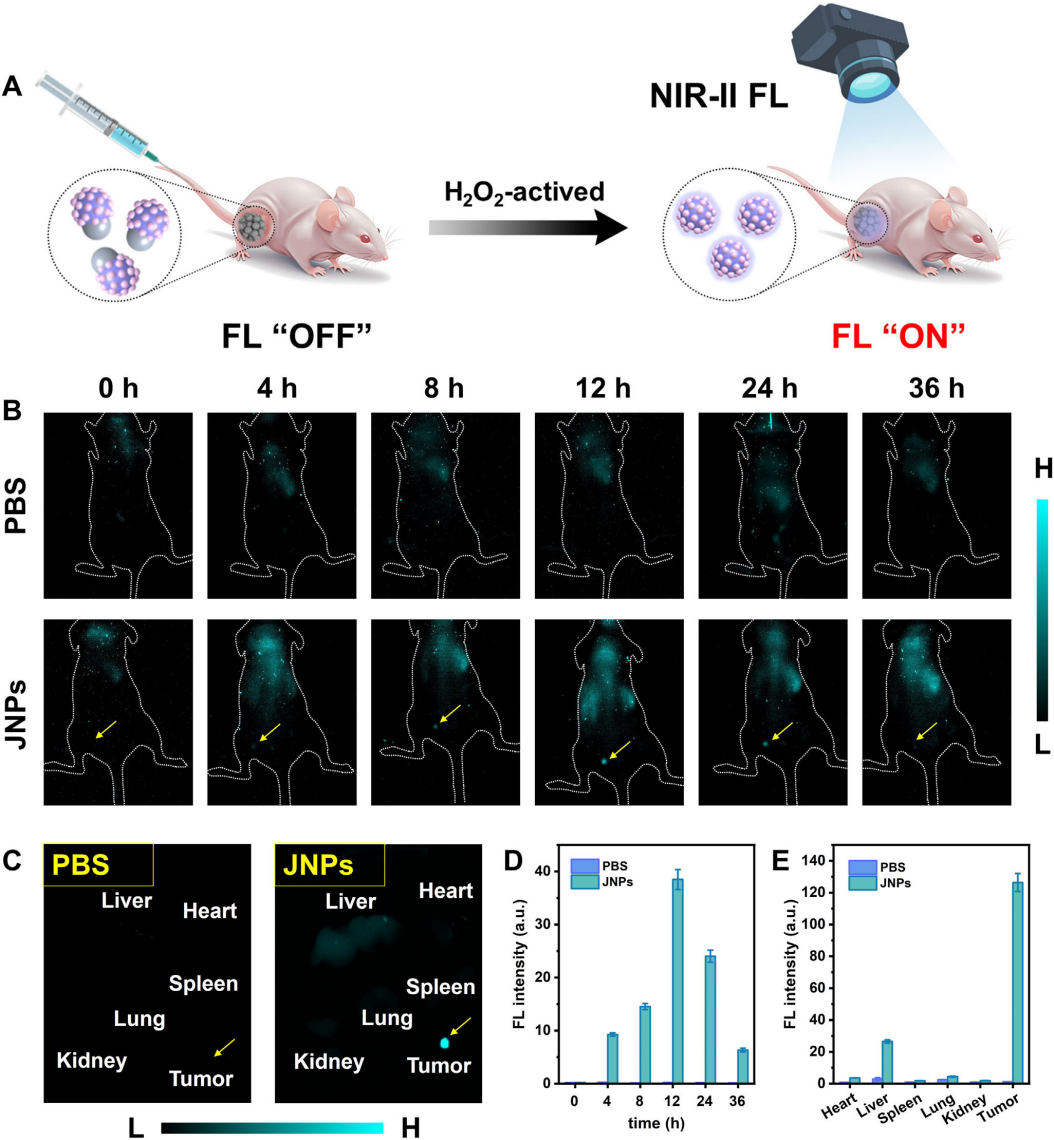
A) Scheme for H_2_O_2_‐activated NIR‐II FL of JNPs enabling in vivo imaging. B) In vivo FL images of tumor‐bearing mice after intravenous injection of PBS and JNPs at different time points. C) NIR‐II FL images of the dissected organs and tumor from the PBS and JNPs‐treated group. D) NIR‐II FL intensity histograms of tumor at different time points. E) FL intensity histograms from the dissected main organs and tumor.

It was well known that H_2_O_2_ is overexpressed in solid tumors compared to normal tissues, which is an important cancer cell biomarker. Taking advantage of the differences in H_2_O_2_ levels between normal tissues and solid tumors as well as the NIR‐II FL switching property of JNPs, we established an endogenous H_2_O_2_‐activated NIR‐II FL imaging method to image subcutaneous tumors (**Figure** **6**A). First, the nude mice were subcutaneously injected with tumor cells to establish a subcutaneous tumor model, and PEG‐modified JNPs (5 mg mL^−1^) were directly injected intratumorally when the tumor volume grew to ≈100 mm^3^. Endogenous H_2_O_2_ at the tumor site could activate the NIR‐II FL of JNPs in situ. The mice were able to rapidly activate strong NIR‐II FL of JNPs within 5 min and outline the location and morphology of subcutaneous tumors. Figure S13 (Supporting Information) shows the NIR‐II FL images of mice at 1250 nm after intratumor injection of JNPs under laser (808 nm) irradiation. The NIR‐II FL signal at the tumor site tended to increase with time and rapidly illuminated the whole tumor within 40 min (Figure S14, Supporting Information). Similarly, after intravenous injection of JNPs into mice, NIR‐II FL at the tumor site was gradually enhanced and reached a maximum at 12 h, while the Control group (PBS group) showed no significant change in FL (Figure 6B,D).

In ex vivo tissue fluorescence imaging, only the tumor site showed significant fluorescence signals, while other major organs exhibited no obvious fluorescence (Figure 6C,E). JNPs that can specifically activate NIR‐II FL by H_2_O_2_ at the tumor site showed excellent tumor diagnostic ability. In addition, specifically activated NIR‐II FL imaging facilitates distinguishing tumor tissue from normal tissue, providing valuable guidance for subsequent precision radiotherapy at the tumor site. Compared with the relative low resolution (∼mm) and accuracy of clinical CT in circling the tumor area, our designed NIR‐II FL imaging probes based on tumor biomarkers of H_2_O_2_ responsiveness are conducive to improving the diagnostic ability of tumors and the accuracy of tumor boundary delineation (∼µm). Precise delineation of the radiotherapy area will reduce damage to normal tissues. Furthermore, the determination of the maximum enrichment of the probe provides the optimal time point for radiotherapy, and JNPs combined with X‐ray maximize radiosensitization.

### 2.6 Enhanced Radiotherapy and Therapy Efficacy Assessment In Vivo

**Figure 7:**
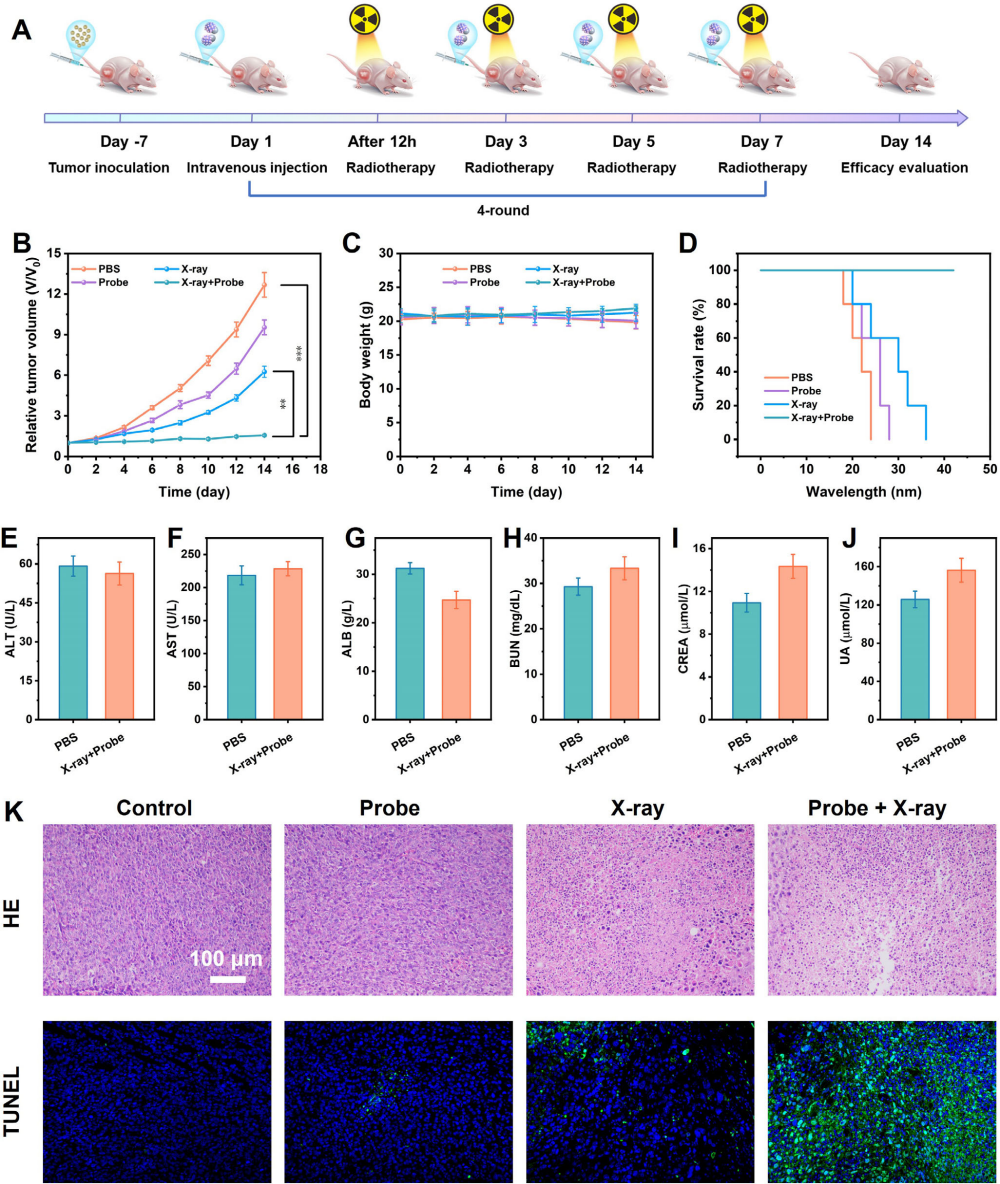
A) Schematic representation of the therapeutic process in which JNPs enhance anti‐tumor effects. The body weight change curves (B), tumor growth curves (C), and relative survival curves (D) of the tumor‐bearing mice after various treatments indicated. (*n* = 5). E–G) The levels of ALT, AST, and ALB in liver function indicators at PBS and probe groups. H–J) The levels of BUN, CREA, and UA in kidney function indicators. K) Representative H&E and TUNEL staining images of tumors from different groups. ***p < 0.001, **p < 0.01.

Encouraged by the outstanding radiosensitization property of JNPs, the radiotherapy effect of JNPs on tumor‐bearing mice was further evaluated. When the tumor volume of the mice reached at ≈80 mm^3^, the mice were randomly divided into four groups to receive different treatment protocols: Control group, Probe group, X‐ray group, and Probe + X‐ray group. The specific operation included administering a 3 Gy dose of radiotherapy to the mice 12 h after intravenous injection of the probe, once every two days, for a total of four times (**Figure** **7**A). The experimental results showed that compared with the Control group, both the Probe group and the X‐ray group inhibited the growth of tumor volume to a certain extent, while the Probe + X‐ray group exhibited the most significant tumor growth inhibition, with tumor volume significantly smaller than that of the other groups (Figure 7B). At the same time, the mice in X‐ray and Probe + X‐ray group showed a slight increase in body weight relative to the Control and Probe group that showed a decrease in body weight, which indicated that JNPs had a high in vivo biosafety as radiosensitizers (Figure 7C). In addition, the median survival time of mice treated with Probe + X‐ray was extended to 40 days, which was significantly higher than that of 20 days in the PBS Control group (Figure 7D). The results of blood biochemical analysis of the probe showed that the liver and kidney functions and hematological indices of the mice were in the normal range, suggesting that the treatment with Probe + X‐ray did not cause obvious inflammation or immune response in the mice (Figure 7E–J; Figure S15, Supporting Information). Histological analysis of the excised major organs showed that the morphological structures of the organs in different treatment groups were normal, further confirming that the Probe + X‐ray treatment had no obvious toxic side effects on the major organs of the mice (Figure S16, Supporting Information). These results strongly confirm that sensitizing radiotherapy based on JNPs enhances the therapeutic effect while maintaining the health status of mice, highlighting the safety and efficacy of this treatment protocol.

To further explore the radio sensitizing radiotherapy mechanism of JNPs, we performed pathological and immunohistochemical analyses of tumor tissues from different treatment groups. Representative hematoxylin and eosin (H&E) staining observed that the necrotic areas of tumor tissues in the Probe + X‐ray group were significantly enlarged and showed more severe damage characteristics compared with other treatment groups. Meanwhile, the enhanced green fluorescence in the TUNEL assay suggested that JNPs could enhance the sensitivity of radiotherapy and effectively promote the apoptosis of tumor cells (Figure 7K). In conclusion, JNPs can be employed as an excellent radiosensitizer in vivo to enhance apoptosis induced by X‐ray, which in turn promotes tumor necrosis and enhances the effect of antitumor therapy.

## 3 Conclusion

In conclusion, we developed a novel kind of selenium‐doped Ag/Ag_2_S Janus nanoparticles (Ag/Ag_2_Se_x_S_y_ JNPs) as a dual‐functional platform for precision cancer activated imaging and radiotherapy (RT). By fine‐tuning selenium doping contents, we optimized the X‐ray absorption, energy storage, and radiosensitization properties of JNPs. DFT calculations confirmed that the doped selenium significantly enhanced X‐ray absorption and energy storage by accelerating electron transfer and lowering the energy barriers thereby increasing the separation efficiency of electron–hole pairs after X‐ray irradiation. Selenium doping optimized the heterostructure of JNPs, making it easier to generate ·OH with high efficiency. The engineering JNPs effectively generate hydroxyl radicals (·OH) upon interaction with tumor‐overexpressed H_2_O_2_ under X‐ray irradiation, inducing apoptosis and amplifying reactive oxygen species (ROS) production for enhanced therapeutic efficacy. Selenium incorporation further improves the electron–hole pair separation and photocurrent response, driving highly efficient ROS generation. Additionally, the H_2_O_2_‐triggered fluorescence recovery enables real‐time NIR‐II fluorescence imaging for precise tumor and RT targeting. Together, these results highlight the Ag/Ag_2_Se_x_S_y_ JNPs as a promising theranostic candidate, integrating real‐time imaging with enhanced radiosensitization for precise and effective cancer treatment.

## 4 Experimental Section

### Preparation of Ag/Ag_2_Se_x_S_y_ JNPs

To prepare Se precursors, Se powder was first dissolved in 50 mL ultrapure (UP) water by mixing it with Na_2_SO_3_ at a molar ratio of 1:3. Sodium selenosulfate (Na_2_SeSO_3_) was then prepared by condensing and refluxing the mixture in a 100 mL round‐bottomed‐flask at 90 °C for 4 h. Afterward, 5 mL of CTAB solution (10 mm), 2.7 mL of AgNO_3_ solution (10 mm), and 5.41 mL of NH_3_·H_2_O (4 mm) were stirred in 50 mL of ultrapure water for 5 min, and then a mixture of TAA and Na_2_SeSO_3_ in different ratios was injected at once. After stirring for 24 h away from light, the products were collected by ultrafiltration centrifugal filter (Mw 10 kDa) and washed three times with UP water to remove the excess CTAB.

### Preparation of PEG‐Modified Ag/Ag_2_Se_x_S_y_ JNPs

1 mL of PEG_2000_‐SH (5 mg) was added dropwise to Ag/Ag_2_Se_x_S_y_ JNPs prepared above and stirred for 12 h. Free PEG_2000_‐SH was then removed by ultrafiltration centrifugal filter (Mw 5 kDa) for two times, and the obtained PEG‐modified Ag/Ag_2_Se_x_S_y_ JNPs were finally re‐dispersed in UP water.

### ESR Detection

ESR spectroscopy was employed to detect the ability of the JNPs to produce ·OH under X‐ray irradiation. 10 µL DMPO solution (a ·OH trapping agent) was taken and mixed with the same concentration of Ag/Ag_2_S, Ag/Ag_2_Se_0.1_S_0.9_, Ag/Ag_2_Se_0.2_S_0.8_, Ag/Ag_2_Se_0.3_S_0.7_ JNPs, and then the signals were detected immediately after X‐ray irradiation (4 Gy).

### H_2_O_2_‐Activated NIR‐II Fluorescence Properties of Ag/Ag_2_Se_x_S_y_ JNPs

Ag/Ag_2_Se_x_S_y_ JNPs solutions were reacted with different concentrations of H_2_O_2_ (1, 5, 10, 25, 50, 100 µM). The NIR‐II FL spectra at the same time points were tested by a fluorescence spectrometer FLS980 (Edinburgh Instruments, England) and the FL images were recorded under 500 ms of exposure time and 3000 mA of current intensity from an in vivo Master small animal NIR‐II bioimaging system equipped with thermoelectric cooled InGaAs camera (Princeton Instruments).

### Specificity Detection of H_2_O_2_ of JNPs

To demonstrate that Ag/Ag_2_Se_x_S_y_ JNPs can be specifically activated by H_2_O_2_, the JNPs were incubated with different types of signaling molecules (HClO, GSH, NaNO_2_, ^1^O_2_, ·OH) for 1 h. Then, the NIR‐II FL intensity of the JNPs was measured under 808 nm laser irradiation.

### Cytotoxicity Assay of JNPs

Cytotoxicity was determined by CCK‐8 assay. The kyse150 cells were inoculated in 96‐well plates. Different concentrations of Ag/Ag_2_Se_0.2_S_0.8_ JNPs were incubated with the cells at 37 °C for 6 h. To evaluate the cell‐killing ability of X‐ray, the cells were also subjected to different doses of X‐ray irradiation (0, 1, 2, 4, 6 Gy) and then continued to be incubated for 12 h. After incubation with 10 µL of CCK‐8 reagent per well for 1 h, the absorbance at 450 nm (OD_450_) was recorded with a Microplate Reader.

### In Vitro H_2_O_2_‐Activated NIR‐II FL Imaging

The cancer cells were inoculated into 96‐well plates and cultured overnight. The JNPs were added to the culture plate and incubated at 37 °C for 6 h. The culture medium was removed and the cells were washed twice with PBS buffer. Then, NIR‐II FL images of the cells with an emission wavelength of 1250 nm were obtained under 808 nm laser irradiation.

### Assessment of DNA Damage After Radiotherapy

DNA damage was assessed by γ‐H2AX Immunofluorescence. Cells were divided into four groups (PBS, Probe, X‐ray, Probe + X‐ray). Cells were selectively incubated with the JNPs for 6 h and subjected to X‐ray irradiation (6 Gy). Then, cells were incubated for another 24 h and washed twice with PBS. Finally, cell images were taken by FL microscopy after staining with a DNA damage kit.

### Animals

BALB/c nude mice (female, 4–6 weeks old) were purchased from GemPharmatech Co., Ltd. (Jiangsu, China). All the animal experiments were approved (No. zryhyy21‐23‐02‐01) by the Institutional Animal Care and Use Committee of China–Japan Friendship Hospital.

### In Vivo Activated NIR‐II FL Imaging

BALB/c nude mice (female, 4–6 weeks old) were purchased from GemPharmatech Co., Ltd. (Jiangsu, China). A subcutaneous tumor of mice model was first constructed. After intravenous injection of 100 µL of Ag/Ag_2_Se_0.2_S_0.8_ JNPs solution into the mice, the in vivo FL images of mice at different time points were recorded using in vivo Master small animal NIR‐II bioimaging system.

### In Vivo Evaluation of Radiotherapy Efficacy

The mice were randomly divided into four groups (PBS, Probe, X‐ray, Probe + X‐ray group). The frequency of radiotherapy was performed every two days for a total of four times. The radiotherapy time point was 12 h after the intravenous injection of Ag/Ag_2_Se_0.2_S_0.8_ JNPs, and the radiation dose was 3 Gy. To evaluate the growth of the tumors, the tumor volume, body weight, and survival time of the mice in each group were monitored, respectively. At the end of the experiment, each organ and tumor tissue of the mouse was collected and analyzed histologically.

## Conflict of Interest

The authors declare no conflict of interest.

## Supporting information



Supporting Information

## Data Availability

The data that support the findings of this study are available in the supplementary material of this article.
