# Supplementary material for: Radio‐Activated Selenium‐Doped Janus Ag/Ag2SexSy Nanoparticles for Precise Cancer NIR‐II Fluorescence Imaging and Radiosensitization Therapy
Source: Adv Sci (Weinh). 2025 Apr 17;12(23):2417828. doi: 10.1002/advs.202417828 (PMC12199479; doi:10.1002/advs.202417828)
Supplement: Supplementary file 1 — Supporting Information [file ADVS-12-2417828-s001.docx]

*Supporting information for:*

**Radio-Activated Selenium-doped Janus Ag/Ag_2_Se_x_S_y_ Nanoparticles for Precise Cancer NIR-II Fluorescence Imaging and Radiosensitization Therapy**

Kang Zhu, Zhanyuan Li, Jingjing Cao, Yixi Cao, Jimei Wang, Shiyu Wang, Ling Chen, Huiqin Zhou, Wei Huang, Hanxun Zou, Qunsheng Li*, Jing Mu*, and Jibin Song*

K. Zhu, J.J. Cao, Y.X, Cao, J.M. Wang, H.Q, Zhou, Prof. Q.S, Li, Prof. J.B. Song

State Key Laboratory of Chemical Resource Engineering, College of Chemistry, College of Chemical Engineering, Beijing University of Chemical Technology, Beijing 100029, China

E-mail: chem64@163.com; liqs@mail.buct.edu.cn

Z.Y. Li, Prof. W. Huang

Shandong Cancer Hospital and Institute, Shandong First Medical University and Shandong Academy of Medical Sciences, Department of Radiation Oncology, Jinan 250117, China

S.Y. Wang, Prof. L. Chen

School of Materials Science and Engineering, University of Jinan, Jinan 250022, China.

Prof. J. Mu

Institute of Precision Medicine, Peking University Shenzhen Hospital, Shenzhen, 518036, P. R. China.

E-mail: jing.mu@pkuszh.com

Prof. H.X. Zou

Fujian Provincial Key Laboratory of Ecology-Toxicological Effects & Control for Emerging Contaminants, Key Laboratory of Ecological Environment and Information Atlas, College of Environmental and Biological Engineering, Putian University, Putian 351100, China,

**Materials**

Cetyltrimethyl ammonium bromide (CTAB), Thioactamide (TAA), Se powder, and sodium sulfite (Na_2_SO_3_) were procured from Sigma Aldrich. Silver nitrate (AgNO_3_), Ammonia solution (NH_3_·H_2_O), and 30% H_2_O_2_ were obtained from Sinopharm Chemical Reagent Co., Ltd (Shanghai, China). Poly (ethylene glycol) methyl ether thiol (PEG_2000_-SH, average Mn 2000) was obtained from Ruixi Biological Technology (Xi’an, China). DMPO was purchased from DOJINDO Laboratories (Shanghai, China). Cell Counting Kit-8 (CCK-8) Kit, Reactive Oxygen Species Assay Kit, Calcein/PI Cell Viability/Cytotoxicity Assay Kit, and DNA Damage Assay Kit were obtained from Beyotime Biotechnology (Shanghai, China). Annexin V-FITC/PI Apoptosis Detection Kit was purchased from Meilunbio (Dalian, China).

**Instruments and Characterizations**

The TEM images of Ag/Ag_2_Se_x_S_y_ JNPs with different Se doping ratios were captured by an HT7700 transmission electron microscope (HITACHI, Japan). NIR-II FL spectra were tested using fluorescence spectrometer FLS980 (Edinburgh Instruments, England) under an external 808 nm laser. NIR-II FL imaging *in vivo* and *in vitro* was captured by *In-Vivo* Master small animal NIR-II bioimaging system (Wuhan Grand-imaging Technology Co., Ltd) equipped with thermoelectric cooled InGaAs camera (Princeton Instruments).

**Ag/Ag_2_Se_x_S_y_ JNPs etching by H_2_O_2_**. In the presence of H_2_O_2_, the Ag portion is gradually etched to form Ag^+^ and then Ag_2_Se_x_S_y_ with electronic defects, thereby restoring the NIR-II FL signal of Ag/Ag_2_Se_x_S_y_ JNPs. To etch the Ag part of Ag/Ag_2_Se_x_S_y_ JNPs, different concentrations of H_2_O_2_ (0-100 μM) were prepared and then incubated with Ag/Ag_2_Se_x_S_y_ JNPs for about 1 h. The NIR-II FL imaging signals of Ag/Ag_2_Se_x_S_y_ JNPs before and after incubation with different concentrations of H_2_O_2_ were recorded at 1250 nm, respectively.

**DFT Calculations** All calculations were carried out for the material in the framework of Density Functional Theory (DFT) using the Vienna Ab initio Simulation Package (VASP 6.3.2). The generalized gradient approximation (GGA) of the Perdew-Burke-Ernzerhof (PBE) function was used to describe the exchange-correlation energy. The projected augmented wave (PAW) method and pseudopotentials were used to describe the interactions between valence electrons and ions. To ensure the efficiency of the computational results and parallel computing. A 3*2*1 k-point grid under Monkhorst-Pack is used in the optimization process and 450 eV truncation energy is set. The lattice parameters and ionic positions of all crystals were fully relaxed, and the convergence criteria for the total energy of all relaxed atoms and the final force were 10^-5^ eV and 0.05 eV/Å, respectively. To correct for the localization effect of D-orbital electrons in transition metal atoms, the Hubbard+U method is used, where the effective U value (Ueff) of Ag is 2.0 eV.

**Hemolysis Assay**

The different concentrations of probes were mixed with 2% erythrocyte suspension and incubated at 37°C for 3 h. The supernatant was taken in a 96-well plate, and the absorbance at 541 nm (OD_541_) was measured by a Microplate Reader, followed by calculating the hemolysis rate.

**Animals**

BALB/c nude mice (female, 4-6 weeks old) were purchased from GemPharmatech Co., Ltd. (Jiangsu, China). All the animal experiments were approved by the Institutional Animal Care and Use Committee of China-Japan Friendship Hospital.

**Statistical Analysis**

All data were presented as mean ± SD (standard deviation). Comparison of the data was conducted with a t-test. Differences were regarded as statistically significant (*P < 0.05, **P < 0.01, ***P < 0.001).


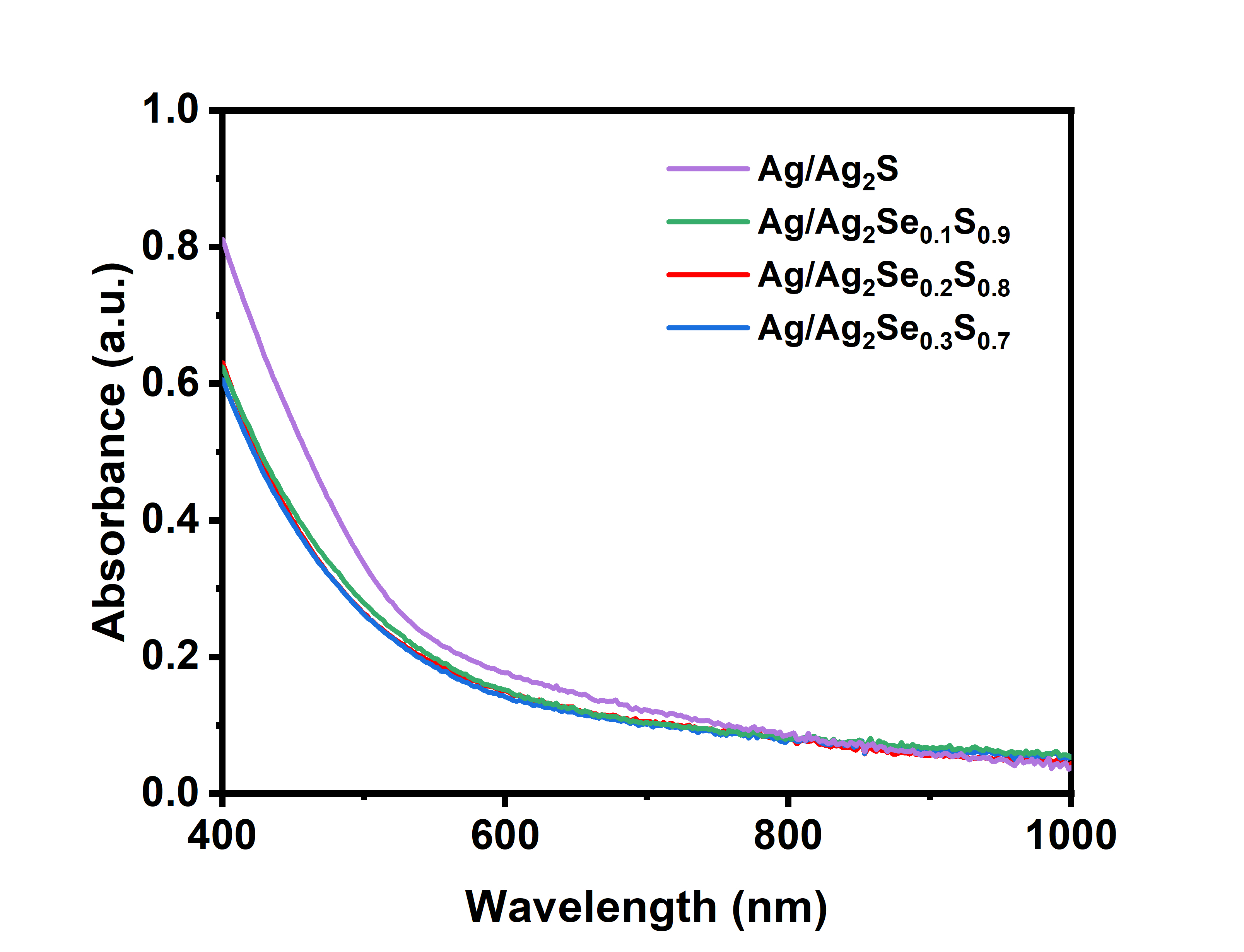


**Figure S1.** UV-vis absorption spectra of Ag/Ag_2_Se_x_S_y_ JNPs with different Se-doping ratios.


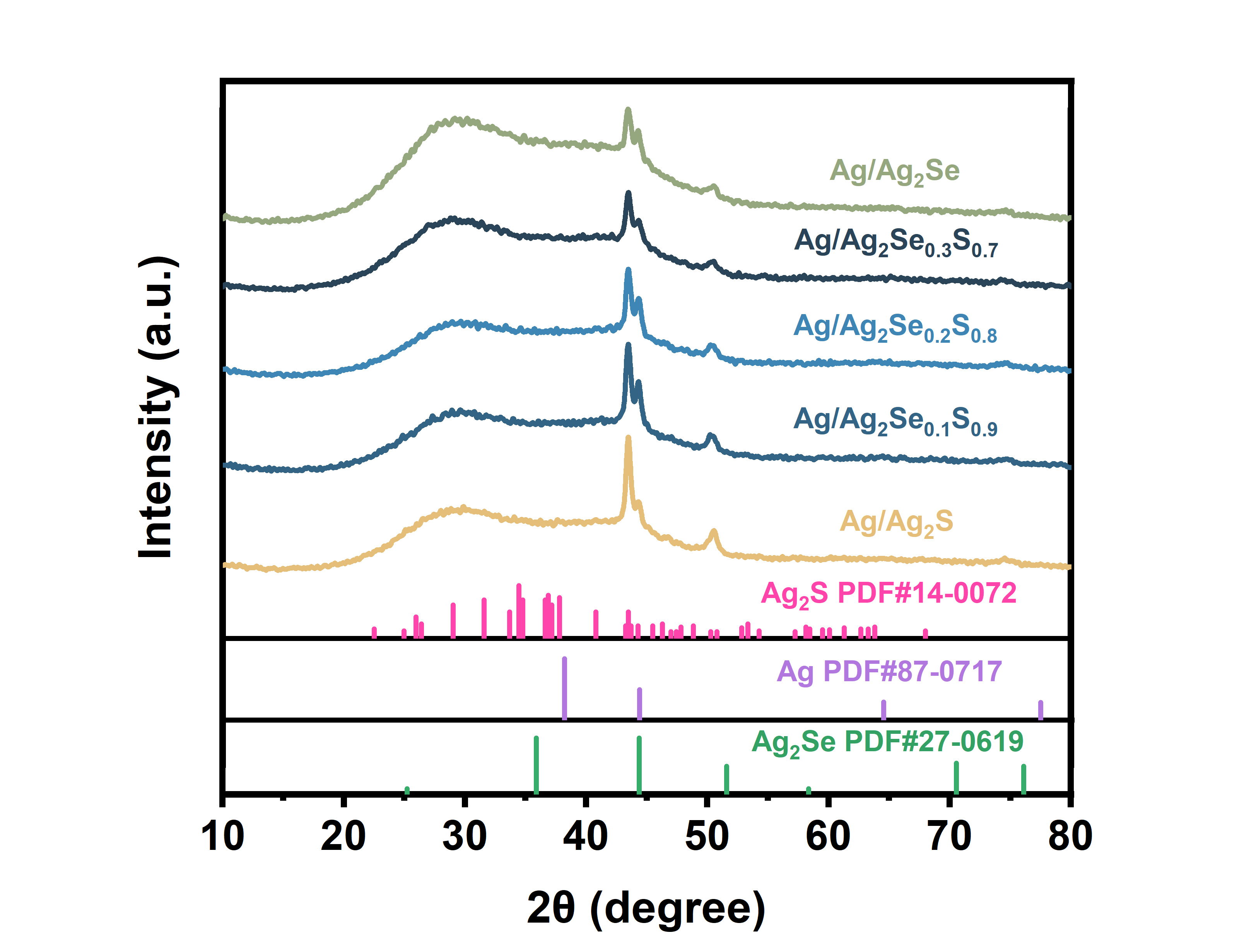


**Figure S2.** X-ray diffraction pattern of Ag/Ag_2_Se_x_S_y_ JNPs with different Se-doping ratios.


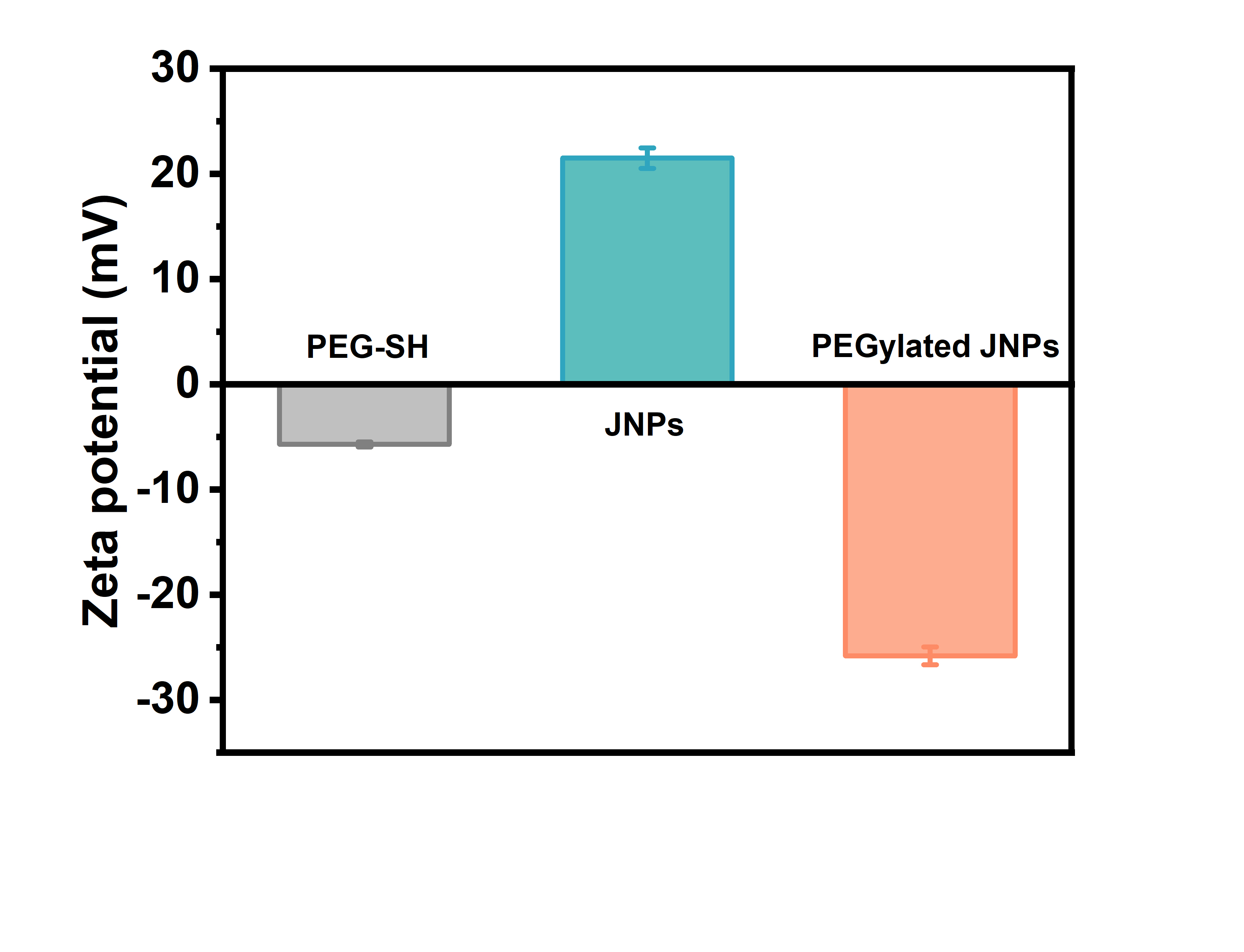


**Figure S3.** Zeta potential measurement of PEG_2000_-SH, Ag/Ag_2_Se_0.2_S_0.8_ JNPs and PEG-modified Ag/Ag_2_Se_0.2_S_0.8_ JNPs.


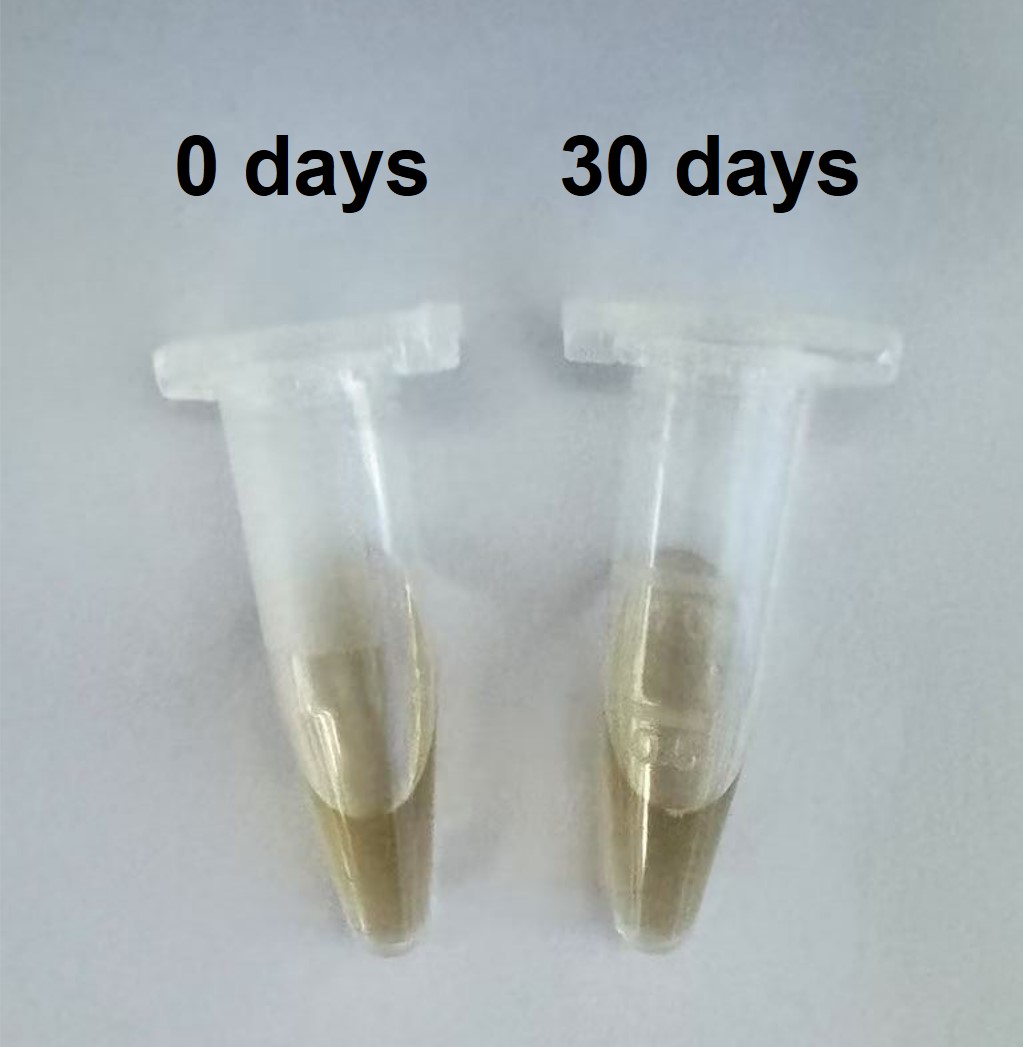


**Figure S4**. Photographs of Ag/Ag_2_Se_0.2_S_0.8_ JNPs stored for 30 days.


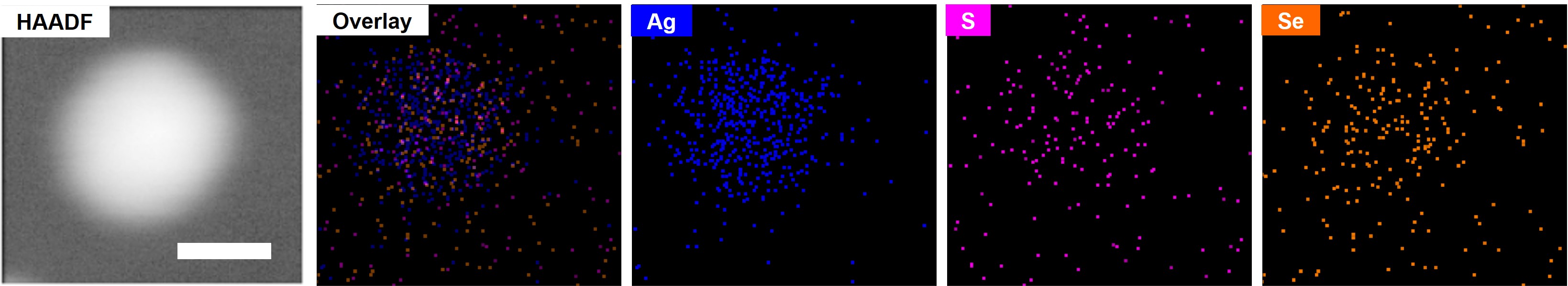


**Figure S5.** High-angle annular dark-field (HAADF) imaging and element mapping of Ag/Ag_2_Se_0.2_S_0.8_ after H_2_O_2_ etching (scale bar: 5 nm).


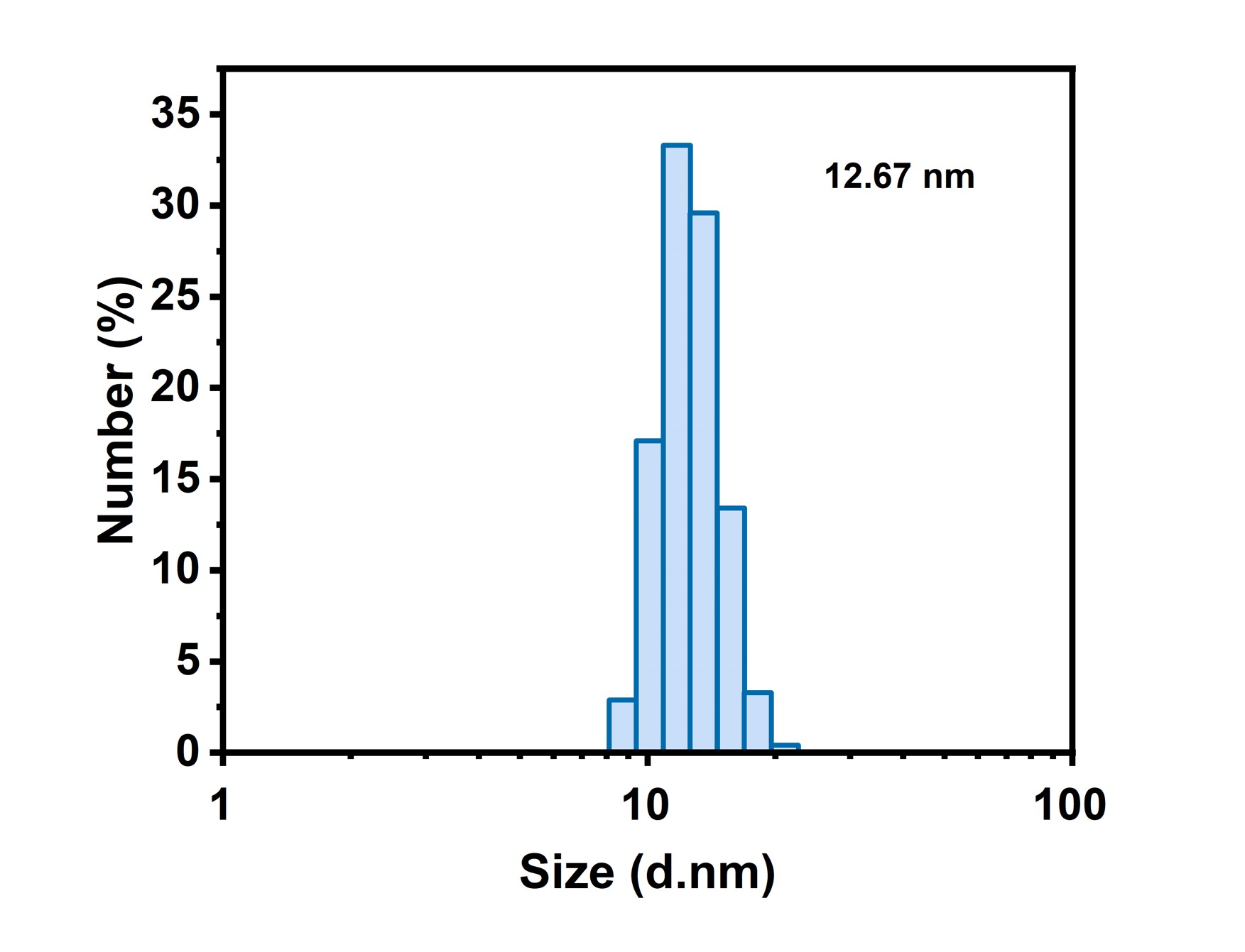


**Figure S6.** Hydrodynamic diameter distributions of Ag/Ag_2_Se_0.2_S_0.8_ after H_2_O_2_ etching.


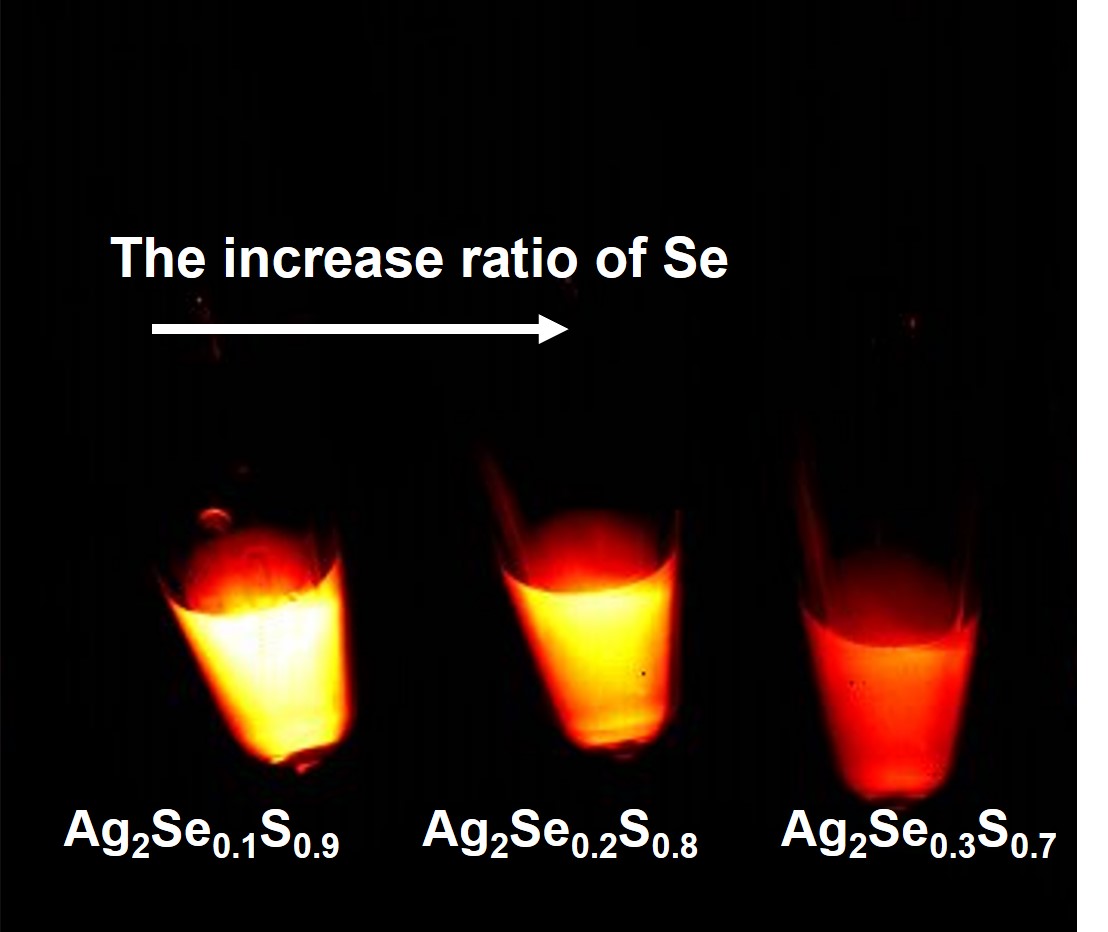


**Figure S7.** NIR-II FL recovery of the same concentration of Ag/Ag_2_Se_x_S_y_ after H_2_O_2_ (100 μM) etching.


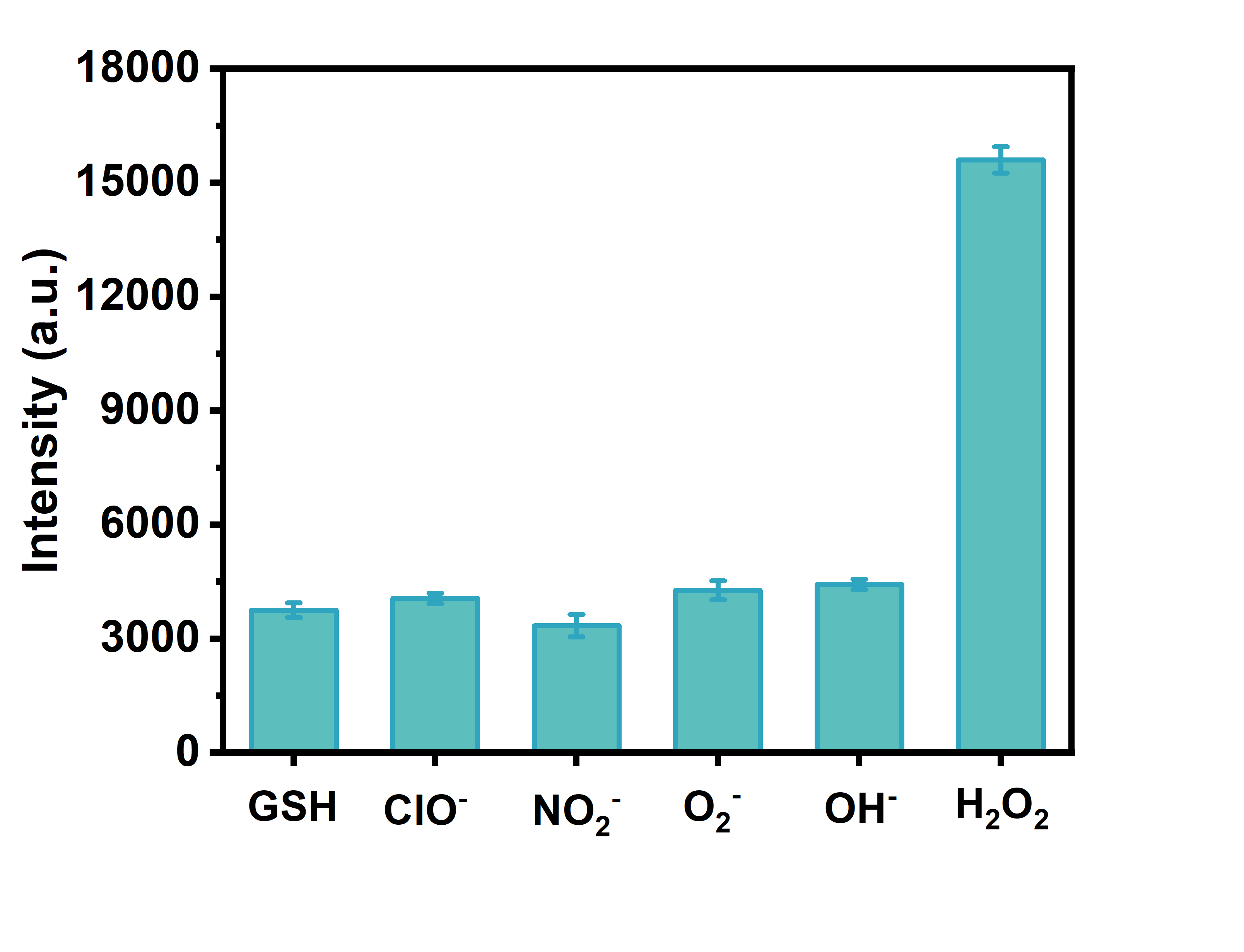


**Figure S8.** The NIR-II FL intensities at 1250 nm of the Ag/Ag_2_Se_0.2_S_0.8_ JNPs incubated with different kinds of ROS.


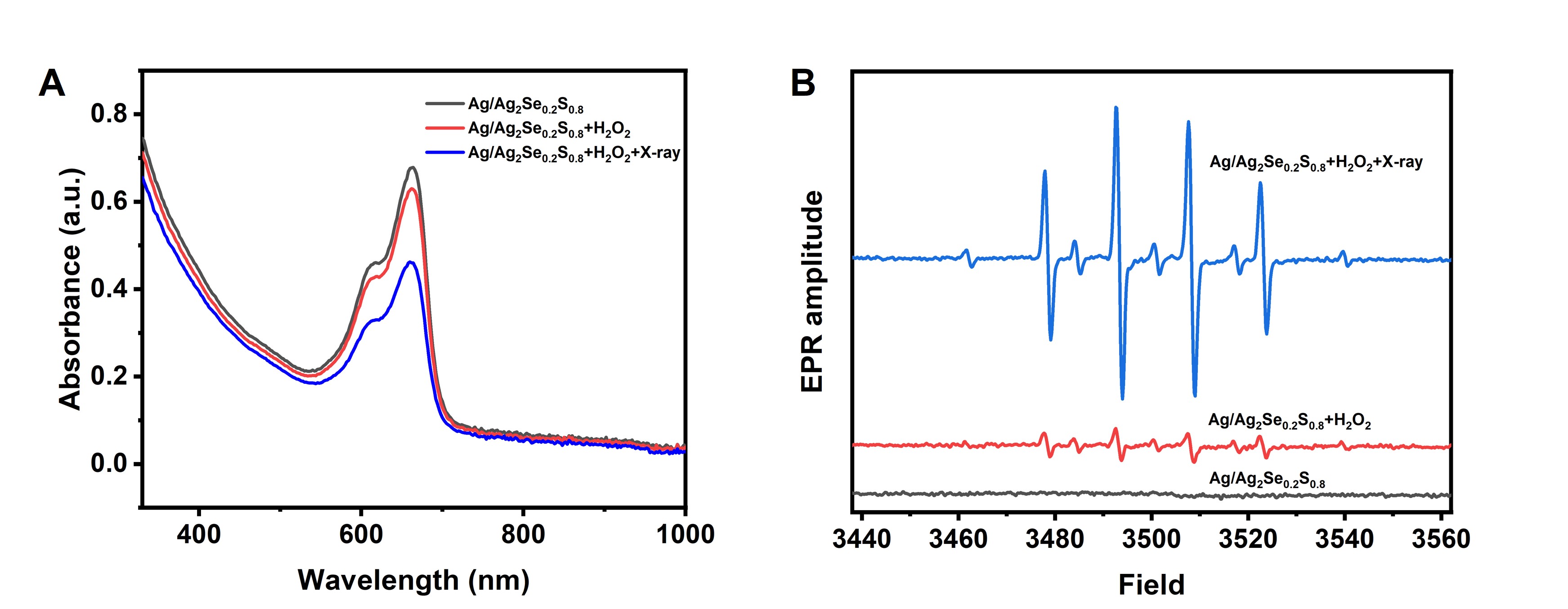


**Figure S9.** (A) The degradation of MB after reacting with Ag/Ag_2_Se_0.2_S_0.8_ and with/without H_2_O_2_ and X-ray. (B) ESR spectroscopy of •OH of Ag/Ag_2_Se_0.2_S_0.8_ with/without H_2_O_2_ and X-ray.


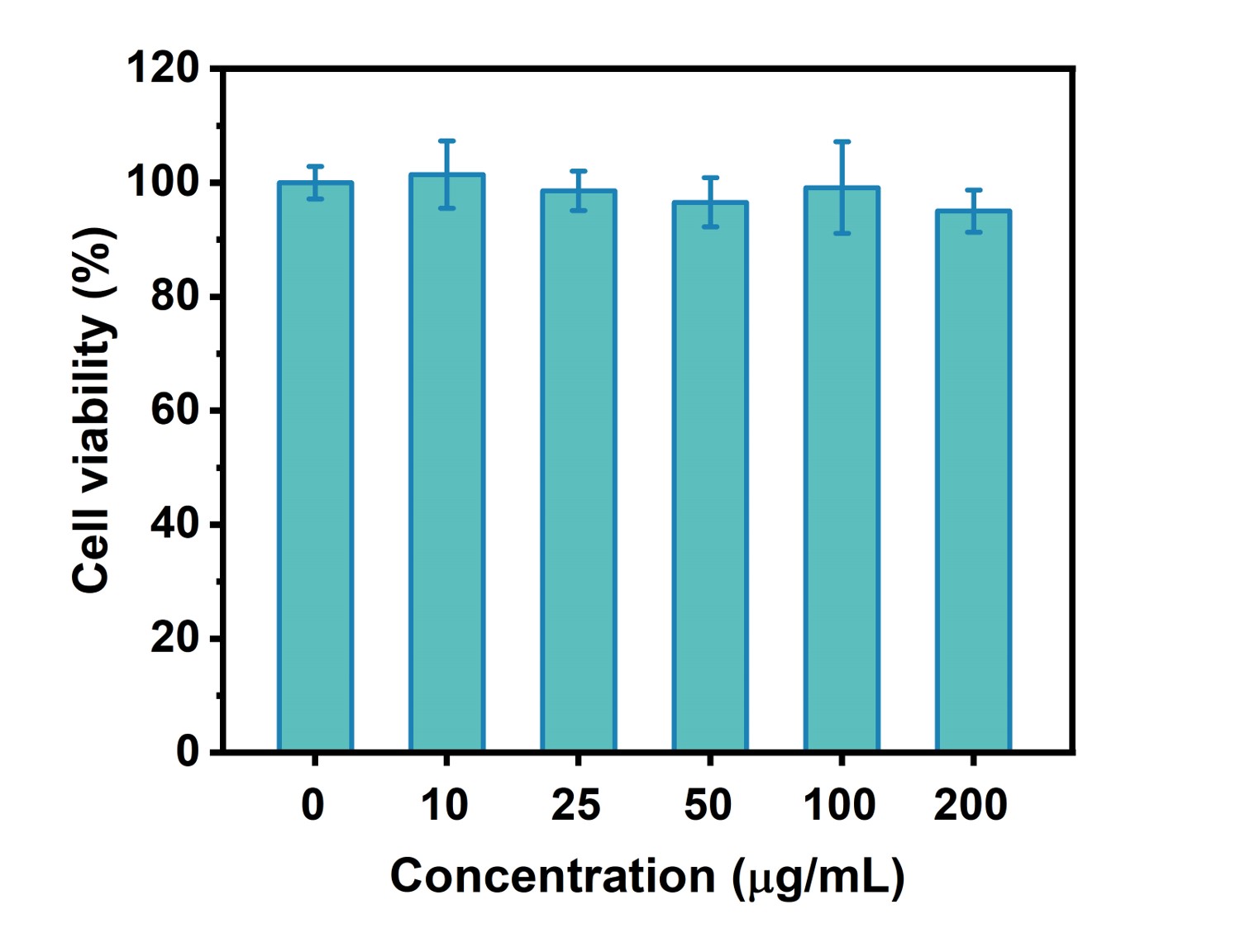


**Figure S10.** Cell viability of cancer cells incubated with different concentrations of Ag/Ag_2_Se_0.2_S_0.8_ JNPs.


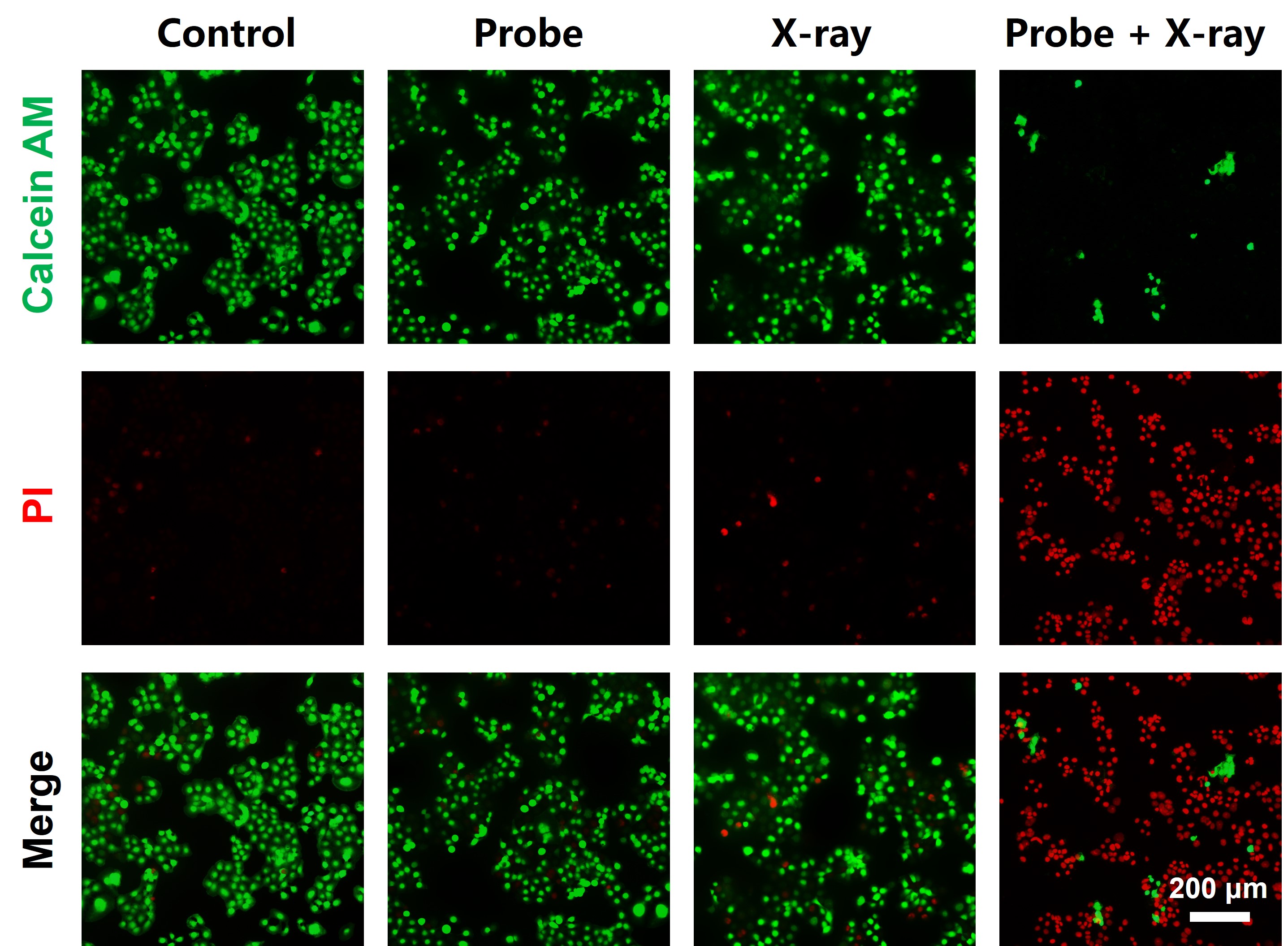


**Figure S11.** FL images of calcein AM (green fluorescence for live cells) and PI (red fluorescence for dead cells) costained cancer cells with different treatments.


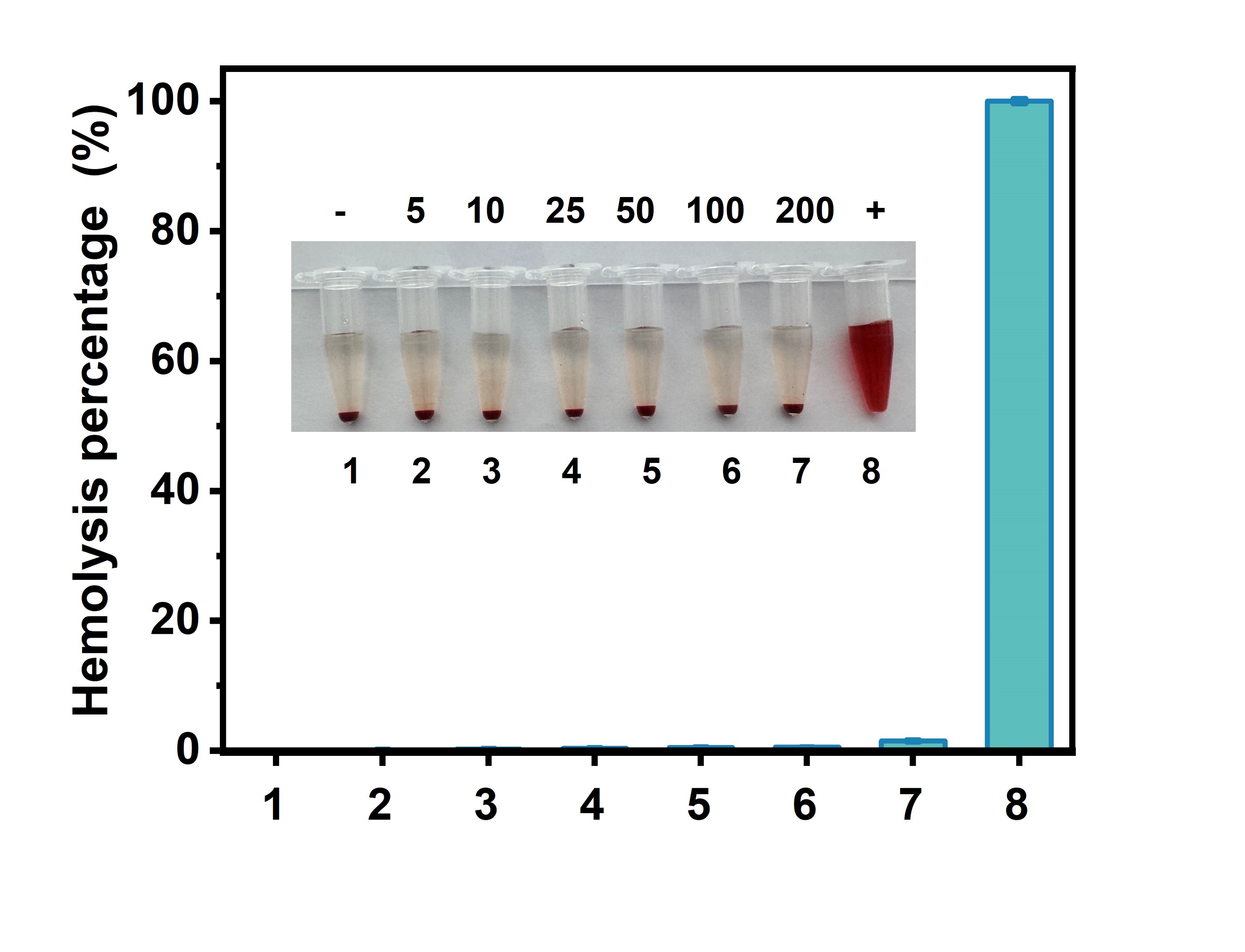


**Figure S12.** Biocompatibility analysis of different concentrations of JNPs, where - and + denote negative and positive controls, respectively.


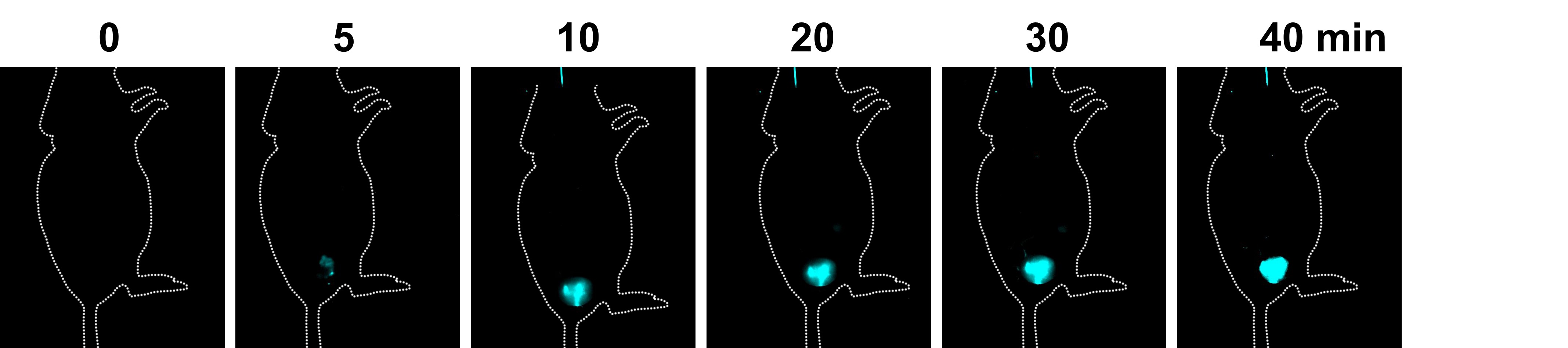


**Figure S13.** *In vivo* FL images of tumor-bearing mice after intratumor injection of Ag/Ag_2_Se_0.2_S_0.8_ at different time points.


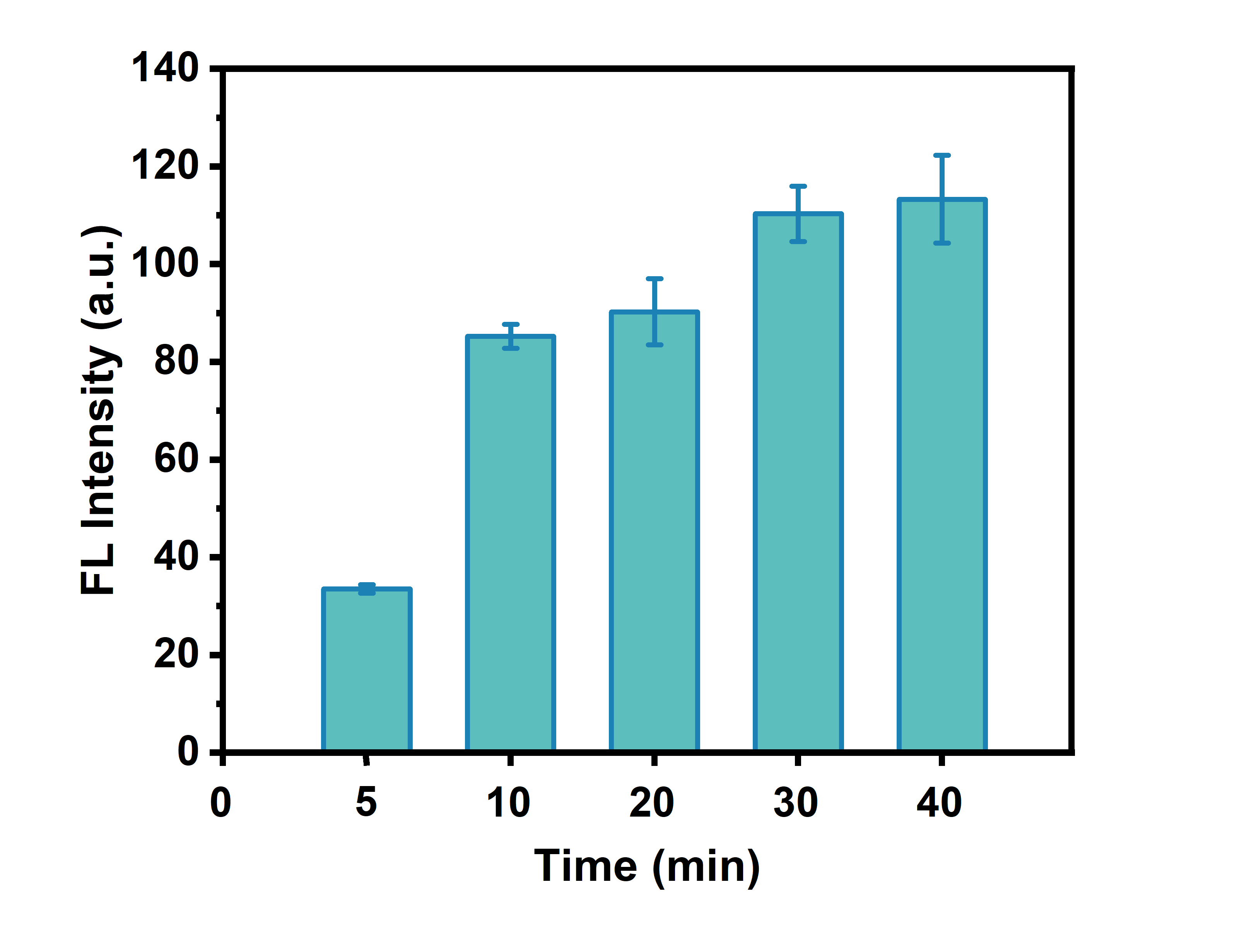


**Figure S14.** Tumor FL intensity histograms of tumor-bearing mice after injection of JNPs at different time points.


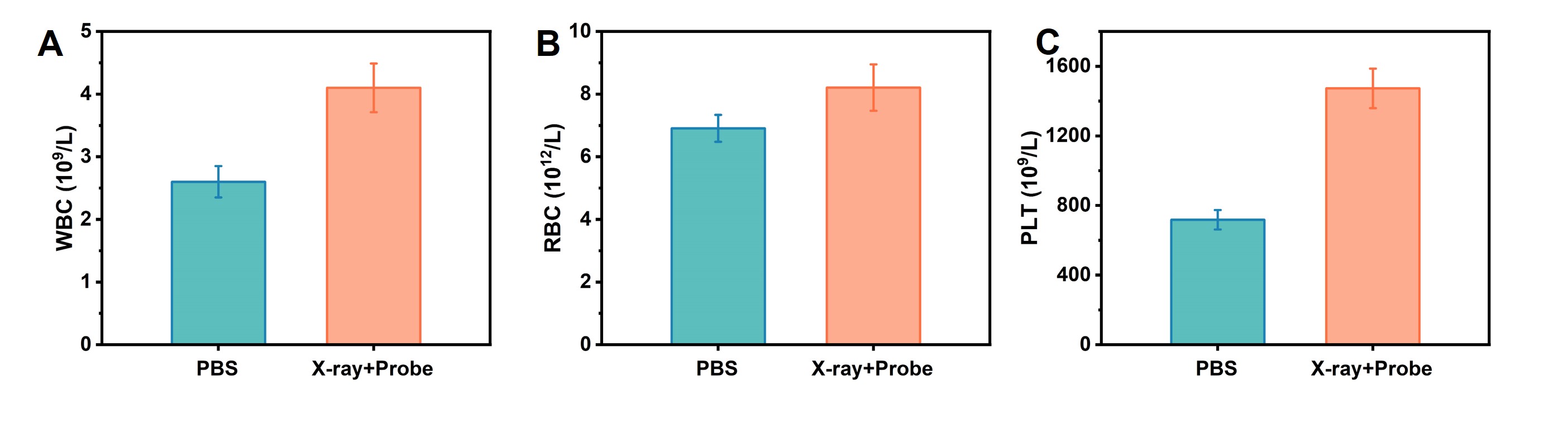


**Figure S15.** The levels of (A) WBC, (B) RBC, and (C) PLT in routine blood tests.


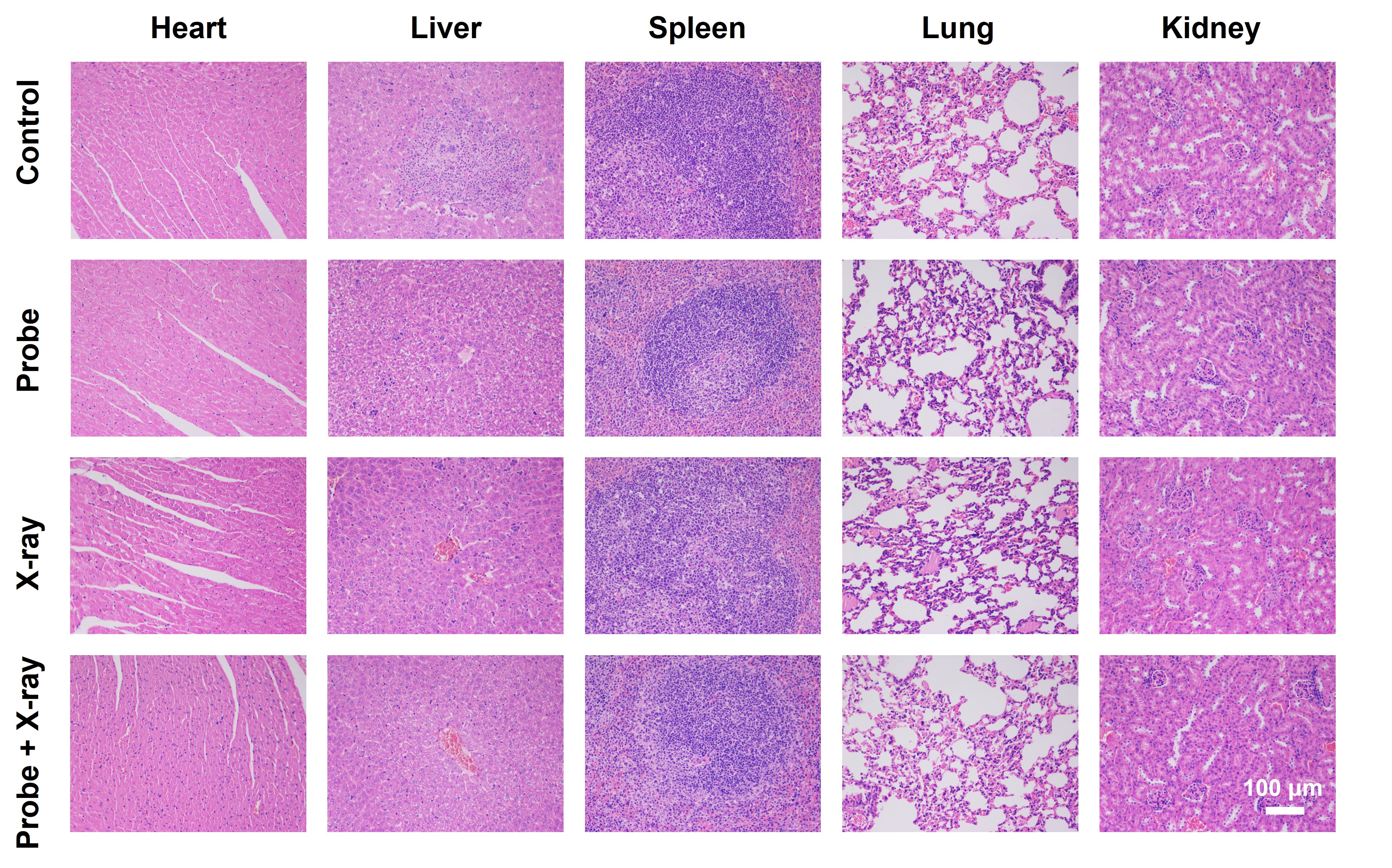


**Figure S16.** H&E staining of the main organs harvested from mice after different treatments (scale bar: 100 μm).
